# Association Between the COVID-19 Pandemic and Insurance-Based Disparities in Mortality After Major Surgery Among US Adults

**DOI:** 10.1001/jamanetworkopen.2022.22360

**Published:** 2022-07-18

**Authors:** Laurent G. Glance, Andrew W. Dick, Ernie Shippey, Patrick J. McCormick, Richard Dutton, Patricia W. Stone, Jingjing Shang, Stewart J. Lustik, Heather L. Lander, Igor Gosev, Karen E. Joynt Maddox

**Affiliations:** 1 Department of Anesthesiology and Perioperative Medicine, University of Rochester School of Medicine, Rochester, New York; 2 Department of Public Health Sciences, University of Rochester School of Medicine, Rochester, New York; 3 RAND Health, RAND, Boston, Massachusetts; 4 Vizient Center for Advanced Analytics, Chicago, Illinois; 5 Memorial Sloan-Kettering Cancer Center, New York, New York; 6 US Anesthesia Partners, Dallas, Texas; 7 Center for Health Policy, Columbia School of Nursing, New York, New York; 8 Department of Surgery, University of Rochester School of Medicine, Rochester, New York; 9 Department of Medicine, Washington University in St Louis, Missouri; 10 Center for Health Economics and Policy at the Institute for Public Health, Washington University in St Louis, Missouri

## Abstract

**Importance:**

The COVID-19 pandemic caused significant disruptions in surgical care. Whether these disruptions disproportionately impacted economically disadvantaged individuals is unknown.

**Objective:**

To evaluate the association between the COVID-19 pandemic and mortality after major surgery among patients with Medicaid insurance or without insurance compared with patients with commercial insurance.

**Design, Setting, and Participants:**

This cross-sectional study used data from the Vizient Clinical Database for patients who underwent major surgery at hospitals in the US between January 1, 2018, and May 31, 2020.

**Exposures:**

The hospital proportion of patients with COVID-19 during the first wave of COVID-19 cases between March 1 and May 31, 2020, stratified as low (≤5.0%), medium (5.1%-10.0%), high (10.1%-25.0%), and very high (>25.0%).

**Main Outcomes and Measures:**

The main outcome was inpatient mortality. The association between mortality after surgery and payer status as a function of the proportion of hospitalized patients with COVID-19 was evaluated with a quasi-experimental triple-difference approach using logistic regression.

**Results:**

Among 2 950 147 adults undergoing inpatient surgery (1 550 752 female [52.6%]) at 677 hospitals, the primary payer was Medicare (1 427 791 [48.4%]), followed by commercial insurance (1 000 068 [33.9%]), Medicaid (321 600 [10.9%]), other payer (140 959 [4.8%]), and no insurance (59 729 [2.0%]). Mortality rates increased more for patients undergoing surgery during the first wave of the pandemic in hospitals with a high COVID-19 burden (adjusted odds ratio [AOR], 1.13; 95% CI, 1.03-1.24; *P* = .01) and a very high COVID-19 burden (AOR, 1.38; 95% CI, 1.24-1.53; *P* < .001) compared with patients in hospitals with a low COVID-19 burden. Overall, patients with Medicaid had 29% higher odds of death (AOR, 1.29; 95% CI, 1.22-1.36; *P* < .001) and patients without insurance had 75% higher odds of death (AOR, 1.75; 95% CI, 1.55-1.98; *P* < .001) compared with patients with commercial insurance. However, mortality rates for surgical patients with Medicaid insurance (AOR, 1.03; 95% CI, 0.82-1.30; *P* = .79) or without insurance (AOR, 0.85; 95% CI, 0.47-1.54; *P* = .60) did not increase more than for patients with commercial insurance in hospitals with a high COVID-19 burden compared with hospitals with a low COVID-19 burden. These findings were similar in hospitals with very high COVID-19 burdens.

**Conclusions and Relevance:**

In this cross-sectional study, the first wave of the COVID-19 pandemic was associated with a higher risk of mortality after surgery in hospitals with more than 25.0% of patients with COVID-19. However, the pandemic was not associated with greater increases in mortality among patients with no insurance or patients with Medicaid compared with patients with commercial insurance in hospitals with a very high COVID-19 burden.

## Key Points

**Question:**

Was the COVID-19 pandemic associated with greater changes in mortality after major surgery among patients with Medicaid insurance or without insurance compared with patients with commercial insurance?

**Findings:**

In this cross-sectional study of 2 950 147 adults undergoing major surgery, mortality rates among patients with Medicaid insurance and patients without insurance did not increase more than the rate among patients with commercial insurance in hospitals with a high COVID-19 burden compared with hospitals with a low COVID-19 burden.

**Meaning:**

These findings suggest that the early phase of the pandemic was not associated with increases in insurance-based disparities in mortality after major surgery.

## Teaser

This cross-sectional study examines whether insurance type was associated with COVID-19 mortality after major surgery among US patients.

## Introduction

The COVID-19 pandemic led to hundreds of thousands of deaths and has created unprecedented disruptions in usual acute care across the US. During the initial COVID-19 surge in early 2020, many hospitals experienced critical shortages of personal protective equipment and supplies and significant crowding. As a result, nearly all US hospitals suspended elective and nonessential procedures, including major operations, such as coronary artery bypass grafting and hip and knee replacements.^1,2,3^

These disruptions to usual surgical care may have disproportionately impacted disadvantaged populations, including those covered by Medicaid or who are uninsured, groups previously demonstrated to have worse surgical outcomes than their insured peers. Before the pandemic, uninsured patients undergoing noncardiac and cardiac surgery had a greater than 50% higher risk of mortality than did patients with private insurance.^4,5^ If the hospitals that typically provide care to patients with Medicaid or uninsured patients were particularly likely to be overwhelmed with COVID-19 or if the patients were particularly likely to have decrements in health during the pandemic, the COVID-19 pandemic may have been associated with a widening of these already stark gaps in surgical outcomes. Furthermore, individuals from racial and ethnic minority groups have been disproportionately affected by COVID-19, accounting for 36% of COVID-19–related deaths and 70% of non–COVID-19–related excess deaths.^6^ Because Black and Hispanic individuals are disproportionately more likely to lack insurance coverage—while 9% of non-Hispanic White individuals lack health insurance, 31% of Hispanic and 15% of non-Hispanic Black individuals are uninsured^7^—any decrements to uninsured populations may worsen gaps in racial and ethnic equity and may have contributed to excess non-COVID mortality.

Our goal in this study was to examine whether the pandemic was associated with a widening of the gap in health outcomes between insured and uninsured individuals undergoing major surgery. COVID-19 created a natural experiment to examine whether hospitals under stress deliver lower-quality care and whether the lower-quality care disproportionately affects uninsured individuals and individuals with Medicaid. We used the wide differences in COVID-19 burden across hospitals (as measured by the proportion of hospitalized patients with COVID-19) to examine the association between the pandemic and surgical outcomes in patients with Medicaid or without insurance compared with patients with commercial insurance.

## Methods

The University of Rochester Research Study Review Board reviewed this study and determined that it met federal and university criteria for exemption because it consisted of secondary research on existing data. Therefore, informed consent was not required. The Strengthening the Reporting of Observational Studies in Epidemiology (STROBE) reporting guideline was used to guide the reporting of this study.^8^

### Data Source

This cross-sectional study was performed using data on patients hospitalized for major surgery from the Vizient Clinical Database, formerly known as the University HealthSystem Consortium. Vizient includes more than 90% of academic medical centers in the US and their affiliated hospitals.^9^ These data have been used in multiple prior studies.^10,11,12,13^ The database contains information on patient demographic characteristics (age, sex, and self-reported race and ethnicity), payer status (commercial insurance, Medicare, Medicaid, uninsured, or other payer), admission status (elective, urgent, or emergency), *International Statistical Classification of Diseases and Related Health Problems, Tenth Revision* diagnostic and procedure codes, encrypted hospital identifiers, all-cause inpatient mortality, and hospital characteristics.

### Study Population

Adult inpatient hospitalizations between January 1, 2018, and May 31, 2020, were included. We grouped operations using the coding algorithm from the Centers for Disease Control and Prevention National Healthcare Safety Network.^14^ We included the following major operations in the analysis: abdominal aortic aneurysm repair; limb amputations; appendix surgery; bile duct, liver, or pancreatic surgery; coronary artery bypass graft surgery; cardiac surgery (eg, aortic valve replacement); carotid endarterectomy; cholecystectomy; colon surgery; craniotomy; spinal fusion; fracture surgery; gastric surgery; knee arthroplasty; hip arthroplasty; laminectomy; peripheral vascular bypass surgery; small-bowel surgery; thoracic surgery; and exploratory laparotomy. We excluded patients with missing data on admission status, age, sex, or race and ethnicity; patients in hospitals with missing information on the proportion of patients who tested positive for COVID-19; and patients from hospitals that were not included in the database in both the baseline period and the first wave of the pandemic (eFigure 1 in the Supplement).

### Statistical Analysis

Our goal was to examine the differential association of the pandemic with outcomes among patients undergoing major surgery as a function of the primary payer status. The outcome of interest was inpatient all-cause mortality. We hypothesized that the pandemic would be associated with increases in surgical mortality and that these increases would be greater for uninsured patients or patients with Medicaid compared with patients with commercial insurance.

We used univariate logistic regression to describe differences in key characteristics of patients by payer status. We analyzed changes in inpatient all-cause mortality before the pandemic (prepandemic period [January 1, 2018, to February 29, 2020]) and during the first wave of the pandemic (surge [March 1 to May 31, 2020]) as a function of the hospital COVID-19 burden. The exposure was the hospital proportion of patients with COVID-19 during the first wave of COVID-19 cases between March 1 and May 31, 2020, stratified as low (≤5.0%), medium (5.1%-10.0%), high (10.1%-25.0%), and very high (>25.0%) (eFigure 2 in the Supplement). We quantified the hospital COVID-19 burden as the maximum proportion of patients in a hospital who tested positive for COVID-19 during the surge period. We estimated a multivariable logistic regression model (model 1):

*f*[*E*(*Y_iht_*)] = *β*_0_ + *β*_1_*Surge* + *β*_2_*COVID_h_* + *β*_3_*Surge* × *COVID_h_* + *β*_4_*X_iht_* + *β*_5_*S_iht_*

where *f* is the logit function, *Y_iht_* is the outcome (live or die) for patient *i* during the surge period (0 for baseline and 1 for first wave of the pandemic) who underwent surgery in hospital *h* with COVID-19 burden *COVID_h_*. *X_iht_* is a vector of patient characteristics (age, sex, admission urgency, Elixhauser comorbidities, and COVID-19 test result), and *S_iht_* is a vector of surgical procedures. We characterized the association between mortality and hospital COVID-19 burden using *β_3_*.

We then analyzed changes in the association between inpatient all-cause mortality and payer status before the pandemic (prepandemic period [January 1, 2018, to February 29, 2020]) and during the first wave of the pandemic (surge [March 1 to May 31, 2020]) for all hospitals regardless of the hospital COVID-19 burden. We estimated a multivariable logistic regression model (model 2):

*f*[*E*(*Y_ijt_*)] = *β*_0_ + *β*_1_*Surge* + *β*_2_*Payer_j_* + *β*_3_*Surge* × *Payer_j_* + *β*_4_*X_ijt_* + *β*_5_*S_ijt_*

where the payer status is specified by *Payer_j_* (commercial insurance, Medicare, Medicaid, other payers, or no insurance). We characterized the relative change in mortality between the baseline period and the surge period as a function of payer status using *β_3_*.

Finally, we examined whether the association between mortality and payer status during the pandemic was a function of the hospital COVID-19 burden using a quasi-experimental triple-difference (DDD) approach. We assumed that patients in hospitals with a very low COVID-19 burden belonged to the untreated group, whereas patients in hospitals with higher COVID-19 burdens belonged to the treatment group. We estimated a multivariable logistic regression model (model 3):

*f*[*E*(*Y_ijht_*)] = *β*_0_ + *β*_1_*Surge* + *β*_2_*Payer_j_* + *β*_3_*COVID_h_* + *β*_4_*Surge* × *Payer_j_* + *β*_5_*Surge* × *COVID_h_* + *β*_6_*Payer_j_* × *COVID_h_* + *β*_7_*Surge* × *COVID_h_* × *Payer_j_* + *β*_8_*X_ijht_* + *β*_9_*S_ijht_*

where *COVID_h_* is the hospital COVID-19 burden, and the other variables are the same as those in models 1 and 2. We characterized the relative change in mortality between the prepandemic period and the surge period for patients with no insurance or with Medicaid vs patients with commercial insurance as a function of the hospital COVID-19 burden using the coefficient for the 3-way interaction term *β_7_*.

We also conducted several sensitivity analyses. First, we reestimated model 1 separately for patients undergoing (1) elective surgery, (2) urgent surgery, and (3) emergency surgery. Second, we reestimated model 2 separately for each stratum of hospital COVID-19 burden as a sensitivity analysis for the DDD model.

In addition to the sensitivity analyses, we performed 2 post hoc analyses in response to reviewer comments after peer review. We used negative binomial regression to estimate changes in surgical case volumes as a function of the hospital COVID-19 burden during the surge for patients undergoing urgent and emergency operations by specifying the period and the hospital COVID-19 burden as main effects and an interaction between period and the hospital COVID-19 burden. We used the log of the number of months in each period as the offset in the negative binomial regression model.

We used logistic regression analysis to estimate whether the proportion of patients with Medicaid or no insurance increased more in hospitals with a high COVID-19 burden compared with hospitals with a low COVID-19 burden during the surge period. We specified the following model (model 4):

*f*[*E*(Y_iht_)] = *β*_0_ + *β*_1_*Surge_t_* + *β*_2_*Urgency_iht_* + *β*_3_*COVID_h_* + *β*_4_*Surge_t_* × *Urgency_iht_* + *β*_5_*Surge* × *COVID_h_* + *β*_6_*Urgency_iht_* × *COVID_h_* + *β*_7_*Surge_t_* × *Urgency_iht_* × *COVID_h_*

where *f* is the logit function, *Y_iht_* is whether a patient had Medicaid or no insurance (coded as 1) and all other payers (coded as 0), *Surge_t_* is the period (baseline or surge), *Urgency_iht_* is the surgical urgency of the procedure for patient *i* in period *t* in hospital *h*, and *COVID_h_* is the hospital COVID-19 burden. We characterized the relative change in the proportion of patients with Medicaid or no insurance in hospitals who underwent urgent or emergency surgery in hospitals with a high COVID-19 burden compared with hospitals with a low COVID-19 burden during the surge period using *β_7_*.

Data management and statistical analyses were performed using Stata SE/MP software, version 17.0 (StataCorp LLC). We used cluster robust variance estimators in the adjusted analyses to account for the clustering of patients within hospitals. All statistical tests were 2-tailed, and *P* < .05 was considered significant.

## Results

We identified 3 038 518 adult inpatient hospitalizations for major surgery at 713 hospitals during the study period. After excluding patients with missing data on admission status (n = 2611), age (n = 5), sex (n = 210), or race and ethnicity (n = 61 344); from hospitals with missing information on the proportion of patients who tested positive for COVID-19 (n = 24 055); and from hospitals that were not included in the database in both the baseline period and the first wave of the pandemic (n = 146), the final analytic data set consisted of 2 950 147 patients in 677 hospitals. Among these 2 950 147 patients (1 550 752 female [52.6%]), the primary payer was Medicare (1 427 791 [48.4%]), followed by commercial insurance (1 000 068 [33.9%]), Medicaid (321 600 [10.9%]), other payer (140 959 [4.8%]), and no insurance (59 729 [2.0%]) (Table 1 and eTable 1 in the Supplement). A total of 202 911 operations (6.9%) occurred during the first wave of the pandemic. A total of 1 414 204 operations (47.9%) were performed in hospitals with a peak COVID-19 burden of 5.0% or less, whereas 607 068 (20.6%) were performed in hospitals with a peak COVID-19 burden of 10.1% to 25.0% and 415 080 (14.1%) in hospitals with a peak COVID-19 burden greater than 25.0%. A total of 1 777 879 operations (60.3%) were elective, whereas 278 239 (9.4%) were urgent and 894 029 (30.3%) were emergency. A total 59 472 patients (2.0%) self-identified as Asian, 381 772 (12.9%) as Black, 2 299 899 (78.0%) as White, and 209 004 (7.1%) as other race and ethnicity.

**Table 1.** Patient Characteristics by Payer Status

Characteristic	Patients, No. (%)					
	Total (N = 2 950 147)	Commercial insurance (n = 1 000 068)	Medicare (n = 1 427 791)	Medicaid (n = 321 600)	Uninsured (n = 59 729)	Other payer (n = 140 959)
Hospitalized with COVID-19, %						
≤5.0	1 414 204 (47.9)	468 163 (46.8)	708 879 (49.7)	141 802 (44.1)	32 403 (54.3)	62 957 (44.7)
5.1-10.0	513 795 (17.4)	161 309 (16.1)	243 337 (17)	57 993 (18)	16 520 (27.7)	34 636 (24.6)
10.1-25.0	607 068 (20.6)	227 763 (22.8)	286 627 (20.1)	63 070 (19.6)	6199 (10.4)	23 409 (16.6)
>25.0	415 080 (14.1)	142 833 (14.3)	188 948 (13.2)	58 735 (18.3)	4607 (7.7)	19 957 (14.2)
Period						
Baseline	2 747 236 (93.1)	935 033 (93.5)	1 329 865 (93.1)	296 969 (92.3)	54 416 (91.1)	130 953 (92.9)
Surge	202 911 (6.9)	65 035 (6.5)	97 926 (6.9)	24 631 (7.7)	5313 (8.9)	10 006 (7.1)
Admission status						
Emergency	894 029 (30.3)	232 370 (23.2)	434 120 (30.4)	137 625 (42.8)	38 445 (64.4)	51 469 (36.5)
Urgent	278 239 (9.4)	82 336 (8.2)	141 150 (9.9)	34 280 (10.7)	7538 (12.6)	12 935 (9.2)
Elective	1 777 879 (60.3)	685 362 (68.5)	852 521 (59.7)	149 695 (46.6)	13 746 (23)	76 555 (54.3)
Sex						
Female	1 550 752 (52.6)	521 922 (52.2)	776 419 (54.4)	174 933 (54.4)	26 221 (43.9)	51 257 (36.4)
Male	1 399 395 (47.4)	478 146 (47.8)	651 372 (45.6)	146 667 (45.6)	33 508 (56.1)	89 702 (63.6)
Age group, y						
18-30	149 731 (5.1)	74 078 (7.4)	4782 (0.3)	47 672 (14.8)	10 326 (17.3)	12 873 (9.1)
31-50	521 567 (17.7)	282 782 (28.3)	52 258 (3.7)	121 455 (37.8)	24 329 (40.7)	40 743 (28.9)
51-64	918 197 (31.1)	531 128 (53.1)	170 981 (12)	137 770 (42.8)	21 532 (36.1)	56 786 (40.3)
65-74	792 220 (26.9)	93 631 (9.4)	664 564 (46.5)	9716 (3)	2321 (3.9)	21 988 (15.6)
75-79	273 891 (9.3)	11 351 (1.1)	254 711 (17.8)	2537 (0.8)	612 (1)	4680 (3.3)
80-84	162 233 (5.5)	4426 (0.4)	153 818 (10.8)	1475 (0.5)	349 (0.6)	2165 (1.5)
85-89	86 588 (2.9)	1879 (0.2)	82 723 (5.8)	664 (0.2)	143 (0.2)	1179 (0.8)
≥90	45 720 (1.6)	793 (0.1)	43 954 (3.1)	311 (0.1)	117 (0.2)	545 (0.4)
Race						
Asian	59 472 (2)	23 072 (2.3)	22 852 (1.6)	9587 (3)	1160 (1.9)	2801 (2)
Black	381 772 (12.9)	110 739 (11.1)	156 203 (10.9)	78 347 (24.4)	11 926 (20)	24 557 (17.4)
White	2 299 899 (78)	802 875 (80.3)	1 183 060 (82.9)	182 522 (56.8)	35 171 (58.9)	96 271 (68.3)
Other^a^	209 004 (7.1)	63 382 (6.3)	65 676 (4.6)	51 144 (15.9)	11 472 (19.2)	17 330 (12.3)
Tested positive for COVID-19 during surge^b^	1969 (0.97)	429 (0.66)	936 (0.96)	440 (1.79)	40 (0.75)	124 (1.24)

^a^ All individuals of races other than White, Black, and Asian were grouped in the other category because the original data were categorized in this manner.

^b^ The surge period was defined as the period from March 1 to May 31, 2020.

Compared with patients with commercial insurance, Medicare, or other payers, patients with Medicaid or no insurance were more likely to undergo urgent (odds ratio [OR], 1.74; 95% CI, 1.72-1.77; *P* < .001) or emergency (OR, 2.42; 95% CI, 2.40-2.44; *P* < .001) surgery; to be Asian (OR, 2.11; 95% CI, 2.07-2.16; *P* < .001), Black (OR, 2.96; 95% CI, 2.94-2.99; *P* < .001), or other race and ethnicity (OR, 4.09; 95% CI, 4.05-4.13; *P* < .001); to test positive for COVID-19 (OR, 2.20; 95% CI, 1.99-2.43; *P* < .001); and to have comorbidities, including paralysis, liver disease, HIV infection, alcohol abuse, drug abuse, and psychosis (eTable 1 in the Supplement). A total of 113 hospitals (16.7%) had 500 or more beds, and 307 (45.3%) had fewer than 100 beds. A total of 346 hospitals (51.1%) had a peak inpatient COVID-19 census of 5.0% or less, and 84 (12.4%) had a peak inpatient COVID-19 census greater than 25.0% (Table 2).

**Table 2.** Hospital Characteristics

Characteristic	Hospitals, No. (%) (N = 677)
Census zone	
Midwest	
East North Central	141 (20.8)
West North Central	81 (12)
Northeast	
Middle Atlantic	102 (15.1)
New England	47 (6.9)
South	
East South Central	15 (2.2)
South Atlantic	115 (17)
West South Central	59 (8.7)
West	
Mountain	59 (8.7)
Pacific	52 (7.7)
Missing data	6 (0.9)
Total beds	
<100	307 (45.3)
100-249	139 (20.5)
250-499	118 (17.4)
≥500	113 (16.7)
COVID-19 burden during surge, %^a^	
≤5.0	346 (51.1)
5.1-10.0	127 (18.8)
10.1-25.0	120 (17.7)
>25.0	84 (12.4)

^a^ The surge period was defined as the period from March 1 to May 31, 2020.

**Figure 1.**
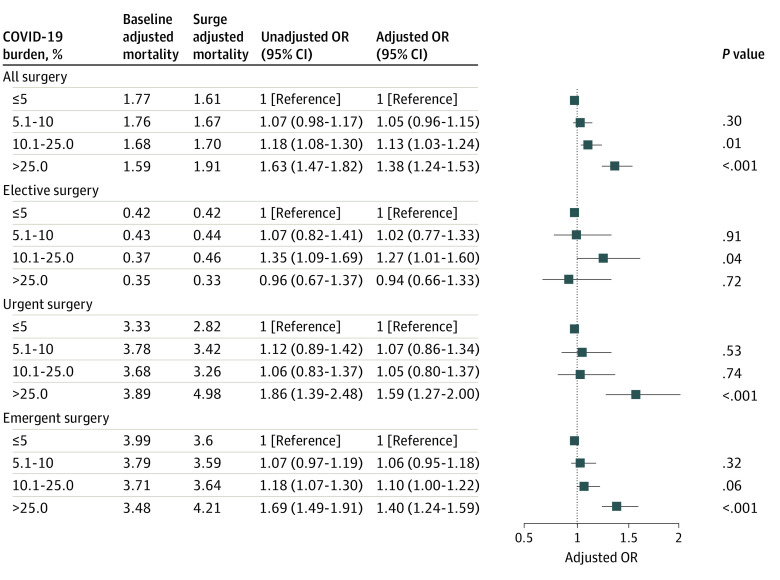
Changes in Mortality Between the Baseline and Surge Periods as a Function of Hospital COVID-19 Burden for All Surgeries and Then Stratified by Surgical Urgency Models were adjusted for age, sex, race and ethnicity, payer status, COVID-19 test results, comorbidities, and surgical procedures. Full models are shown in eTable 2 in the Supplement. Squares indicate adjusted odds ratios (ORs), with horizontal lines indicating 95% CIs.

Mortality rates increased more among patients undergoing surgery during the first wave of the pandemic in hospitals with high (adjusted OR [AOR], 1.13; 95% CI, 1.03-1.24; *P* = .01) and very high (AOR, 1.38; 95% CI, 1.24-1.53; *P* < .001) COVID-19 burdens compared with patients in hospitals with a low COVID-19 burden (Figure 1 and eTable 2 in the Supplement). Mortality rates increased more for urgent (AOR, 1.59; 95% CI, 1.27-2.00; *P* < .001) and emergency surgery (AOR, 1.40; 95% CI, 1.24-1.59; *P* < .001) than for elective surgery (AOR, 0.94; 95%, 0.66-1.33; *P* = .72) in hospitals with very high COVID-19 burdens compared with hospitals with a low COVID-19 burden (Figure 1 and eTable 2 in the Supplement).

**Figure 2.**
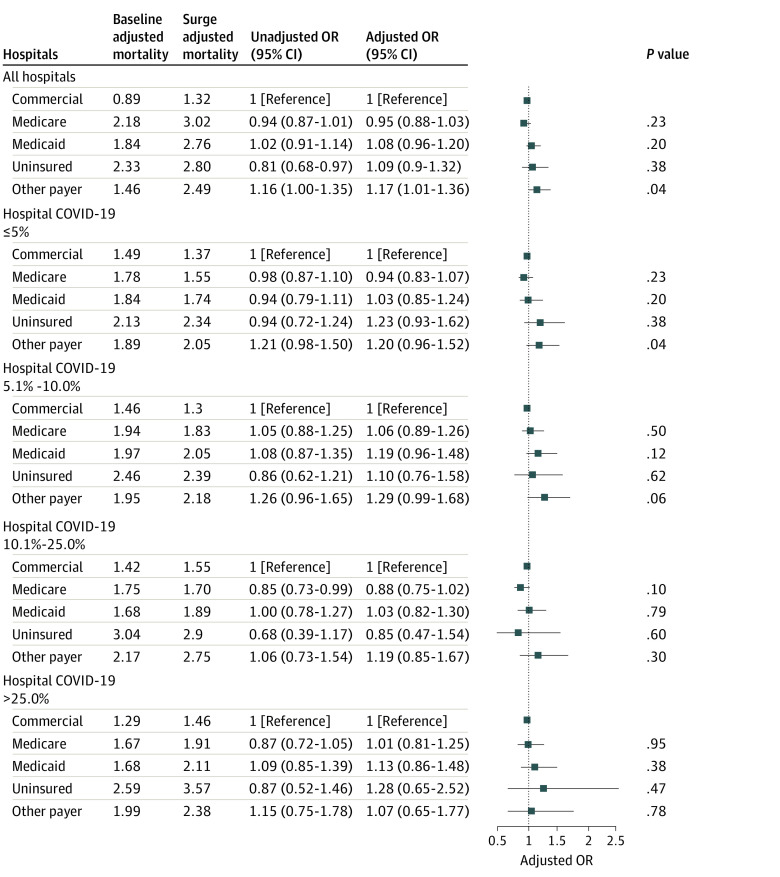
Changes in Mortality Between the Baseline and Surge Periods as a Function of Payer Status for All Hospitals and Then Stratified by Hospital COVID-19 Burden Models were adjusted for age, sex, COVID-19 test results, comorbidities, and surgical procedures. Full models are shown in eTable 3 in the Supplement. Squares indicate adjusted odds ratios (ORs), with horizontal lines indicating 95% CIs.

Patients with Medicaid insurance had 29% higher odds of death (AOR, 1.29; 95% CI, 1.22-1.36; *P* < .001) and patients without insurance had 75% higher odds of death (AOR, 1.75; 95% CI, 1.55-1.98; *P* < .001) compared with patients with commercial insurance (eTable 3 in the Supplement). Mortality rates did not increase more during the first wave of the pandemic among patients with Medicaid (AOR, 1.08; 95% CI, 0.96-1.20; *P* = .20) or without insurance (AOR, 1.09; 95% CI, 0.90-1.32; *P* = .38) compared with patients with commercial insurance (Figure 2 and eTable 3 in the Supplement).

On the basis of the DDD analysis in which we examined the differential association by payer of the first wave of the pandemic with outcomes among patients undergoing surgery in hospitals with high vs low COVID-19 burdens, there was no evidence that patients with Medicaid insurance or no insurance were more adversely affected by undergoing surgery in hospitals with a high COVID-19 burden compared with patients with commercial insurance (joint test for triple interaction term: *P* = .94). The results of the baseline model (model 2) stratified by hospital COVID-19 burden are given in Figure 2 and eTable 3 in the Supplement, and the results of the DDD model are given in eTable 4 in the Supplement. The mortality rate among patients with Medicaid (AOR, 1.03; 95% CI, 0.82-1.30; *P* = .79) or without insurance (AOR, 0.85; 95% CI, 0.47-1.54; *P* = .60) did not increase more than that among patients with commercial insurance in hospitals with a high COVID-19 burden compared with hospitals with a low COVID-19 burden (Figure 2 and eTable 3 in the Supplement). These findings were similar in hospitals with very high COVID-19 burdens. We tested the parallel trend assumption in the baseline period by including a continuous variable for month and interacting it with payer and with the hospital COVID-10 burden and did not find any evidence of a violation of the parallel trend assumption.

In our first post hoc analysis, we found that compared with the baseline period, emergency surgery case volumes were more reduced in hospitals with high (incident rate ratio, 0.88; 95% CI, 0.86-0.90; *P* < .001) and very high (incident rate ratio, 0.82; 95% CI, 0.80-0.84; *P* < .001) COVID-19 burdens compared with hospitals with very low COVID-19 burdens. We found similar results for urgent operations in hospitals with high (OR, 0.92; 95% CI, 0.88-0.95; *P* < .001) and very high (OR, 0.66; 95% CI, 0.63-0.70; *P* < .001) COVID-19 burdens (eFigure 3 in the Supplement).

In our second post hoc analysis, we found that the proportion of patients with Medicaid or without insurance undergoing emergency surgery increased more in hospitals with a very high COVID-19 burden (OR, 1.13; 95% CI, 1.01-1.28; *P* = .04) but not in hospitals with a high COVID-19 burden (OR, 1.11; 95% CI, 0.99-1.24; *P* = .07) compared with hospitals with a low COVID-19 burden. We found no evidence of increases in the proportion of patients with Medicaid or without insurance undergoing urgent surgery during the surge period (eFigure 4 in the Supplement).

## Discussion

In this cross-sectional study of 2 950 147 patients undergoing major inpatient surgical procedures at 677 US hospitals, we found that the odds of mortality in hospitals with a very high COVID-19 burden increased 38% more during the first wave of the pandemic than in hospitals with a low COVID-19 burden. These odds were greater among patients requiring urgent and emergency surgery than among those requiring elective surgery. Patients with Medicaid or no insurance had 29% and 75% higher odds of mortality, respectively, than patients with commercial insurance. However, patients who lacked insurance or had Medicaid did not experience greater changes in mortality during the first wave of the pandemic compared with patients with commercial insurance. These findings suggest that the pandemic did not exacerbate the mortality gap between patients with no insurance or Medicaid and patients with commercial insurance.

The effect of COVID-19 on the US health care system is unprecedented. Surges in hospital admissions have led to shortages of hospital beds, critical care unit beds, ventilators, personal protective equipment, and staff availability.^15^ Overcrowding is associated with increased COVID-19 mortality and possibly with excess non–COVID-19 deaths.^15,16,17^ In response to the first wave of the pandemic, the Centers for Medicare & Medicaid Services recommended that all elective and nonessential operations be delayed to free health care resources and staff to care for patients with COVID-19.^18^ Our study found that despite the substantial reduction in surgical case volumes,^19^ increases in overall hospital COVID-19 caseloads were associated with worse outcomes in surgical patients, especially in those undergoing urgent and emergency surgery. Whether this finding was associated with patients presenting later in their acute illness because of heightened fear of contracting COVID-19 at their local hospital or whether it suggests a higher level of care disruption in hospitals with a higher COVID-19 burden is unclear, and it is likely that both factors contributed to some degree. However, this increase in mortality did not disproportionately affect disadvantaged patients with no insurance or Medicaid. This finding is particularly important in light of the disproportionate effects of COVID-19 on individuals from racial and ethnic minority groups, many of whom lacked insurance or had Medicaid instead of commercial insurance.^20^

Our study supports extensive and longstanding evidence that uninsured individuals or individuals with Medicaid have worse health outcomes than patients with commercial insurance.^4,5,21,22,23,24^ However, it is difficult to disentangle the extent to which these worse outcomes are associated with lower-quality medical care or whether the lack of insurance or Medicaid serves as a proxy for worse health status. The pandemic can serve as a natural experiment to help understand the association of the health care system with this disparity in outcomes. If the health care system contributed to disparities in surgical outcomes through discrimination against uninsured or Medicaid-insured patients, one would expect that the increases in mortality during the pandemic would be worse in these groups because hospitals under stress would allocate their limited resources to more advantaged individuals. Using quasi-experimental methods, our study suggests that although inpatient surgical outcomes worsened during the pandemic, patients with or without insurance were affected equally. Because our study was limited to inpatient mortality, it did not examine the differential association of the pandemic with the health of individuals with low income before they were admitted for surgery. Our study also did not examine the association of the pandemic with postdischarge outcomes among patients with low income. Nonetheless, during the acute care hospitalization, insurance-based disparities did not worsen. However, given the marked inequities in surgical outcomes that we and others have reported,^25^ these findings should not cause health care policy makers or frontline clinicians to be complacent about the health care challenges that individuals with low income experience before and after they leave the hospital. Moreover, the health care challenges experienced by individuals with low income may continue to increase because of ongoing job losses and continued exposure to COVID-19,^26^ which may further exacerbate economic disparities and disrupt insurance coverage. It will be important to track the long-term effects of delays and disruptions to surgical care among disadvantaged groups even after the COVID pandemic has waned.

### Limitations

This study has several important limitations. First, we used inpatient mortality instead of 30-day mortality. It is possible that hospitals discharged patients sooner during the pandemic to increase bed availability for patients with COVID-19 and that patients without insurance or with Medicaid were more likely to be discharged sooner compared with patients with commercial insurance and thus were less likely to die in the hospital. Therefore, increases in inpatient mortality among patients without insurance or with Medicaid during the pandemic could have been masked by earlier hospital discharges. Second, some patients lost employment-based insurance during the pandemic and may have become uninsured or obtained Medicaid coverage. As a result, the pool of uninsured individuals or individuals with Medicaid may have included healthier patients who lost their employer-sponsored insurance during the pandemic. However, the rate of decline in employer-sponsored insurance during the pandemic (0.2 percentage points each week) was likely too small to bias our findings.^27^ Third, we did not examine complication outcomes because of the systematic underreporting of postoperative complications in administrative data.^28,29^ Fourth, our findings may not be generalizable because they are based on a sample of US hospitals. However, our study is the largest multicenter study to date to examine the association of COVID-19 with surgical outcomes and includes a broadly representative set of hospitals. Fifth, our findings in this retrospective study should be interpreted cautiously because observational studies are subject to unmeasured confounding, especially when based on administrative data. In particular, mortality rates in hospitals with the highest burden of patients with COVID-19 may have increased compared with hospitals with the lowest burden of patients with COVID-19 in part because hospitals with high COVID-19 burden limited admissions to patients who were sicker compared with hospitals with low COVID-19 burden. In particular, we found that hospitals with the highest COVID-19 burden experienced greater reductions in urgent and emergency operations compared with hospitals with the lowest COVID-19 burden. We also found that the proportion of patients with Medicaid or without insurance undergoing emergency surgery increased slightly in hospitals with very high COVID-19 burdens during the surge compared with hospitals with a low COVID-19 burden. These findings suggest that hospitals under the greatest stress may have preferentially operated on the sickest patients, which could partly account for the higher mortality rates in hospitals with the highest COVID-19 burdens.

## Conclusions

In this cross-sectional study, the first wave of the COVID-19 pandemic was associated with a higher risk of mortality after surgery in hospitals with a very high burden of COVID-19–positive patients. Although patients with Medicaid or without health insurance were more likely to die after surgery compared with patients with commercial insurance, the pandemic was not associated with greater increases in mortality among these patients than among patients with commercial insurance. Improving outcomes in economically disadvantaged populations may require a greater emphasis on improving the general health of patients from these populations before surgery to narrow the mortality gap after surgery between economically disadvantaged patients and patients with commercial insurance.

## Notes

Supplement. **eFigure 1.** Flow Diagram Describing Selection of Cases in the Analytic Cohort **eFigure 2.** Distribution of Hospital COVID-19 Burden **eFigure 3.** Emergent and Urgent Surgical Case Volumes Stratified by Hospital COVID-19 Burden **eFigure 4.** Changes in the Proportion of All Patients Undergoing (A) Emergency Surgery and (B) Urgent Surgery Who Have Either Medicaid or No Insurance **eTable 1.** Patient Characteristics **eTable 2.** Coefficients for Model Examining the Association Between Changes in All-Cause Inpatient Mortality and Hospital COVID-19 Burden **eTable 3.** Coefficients for Models Examining the Association Between Changes in All-Cause Inpatient Mortality and Payer Status and Hospital COVID-19 Burden **eTable 4.** Coefficients for DDD Model Examining the Association Between Changes in All-Cause Inpatient Mortality and Payer Status and Hospital COVID-19 Burden

## References

[1] Prasad NK, Englum BR, Turner DJ, . A nation-wide review of elective surgery and COVID-surge capacity. J Surg Res. 2021;267:211-216. doi:10.1016/j.jss.2021.05.028 34157490 PMC8213966

[2] Pirracchio R, Mavrothalassitis O, Mathis M, Kheterpal S, Legrand M. Response of US hospitals to elective surgical cases in the COVID-19 pandemic. Br J Anaesth. 2021;126(1):e46-e48. doi:10.1016/j.bja.2020.10.013 33187635 PMC7572110

[3] Nguyen TC, Thourani VH, Nissen AP, . The effect of COVID-19 on adult cardiac surgery in the United States in 717 103 patients. Ann Thorac Surg. 2022;113(3):738-746. doi:10.1016/j.athoracsur.2021.07.015 34343473 PMC8325556

[4] LaPar DJ, Bhamidipati CM, Mery CM, . Primary payer status affects mortality for major surgical operations. Ann Surg. 2010;252(3):544-550. doi:10.1097/SLA.0b013e3181e8fd75 20647910 PMC3071622

[5] LaPar DJ, Stukenborg GJ, Guyer RA, . Primary payer status is associated with mortality and resource utilization for coronary artery bypass grafting. Circulation. 2012;126(11)(suppl 1):S132-S139. doi:10.1161/CIRCULATIONAHA.111.083782 22965973 PMC3448930

[6] Cronin CJ, Evans WN. Excess mortality from COVID and non-COVID causes in minority populations. Proc Natl Acad Sci U S A. 2021;118(39):e2101386118. doi:10.1073/pnas.2101386118 34544858 PMC8488621

[7] Cohen RAMM, Cha AE, Terlizzi EP. Health insurance coverage: early release of estimates from the National Health Interview Survey, January-June 2021. National Center for Health Statistics. 2021. Accessed December 29, 2021.https://www.cdc.gov/nchs/data/nhis/earlyrelease/insur202111.pdf

[8] von Elm E, Altman DG, Egger M, Pocock SJ, Gøtzsche PC, Vandenbroucke JP; STROBE Initiative. The Strengthening the Reporting of Observational Studies in Epidemiology (STROBE) statement: guidelines for reporting observational studies. Lancet. 2007;370(9596):1453-1457. doi:10.1016/S0140-6736(07)61602-X 18064739

[9] Loehrer AP, Chang DC, Scott JW, . Association of the Affordable Care Act Medicaid expansion with access to and quality of care for surgical conditions. JAMA Surg. 2018;153(3):e175568. doi:10.1001/jamasurg.2017.5568 29365029 PMC5885934

[10] Saad M, Kennedy KF, Imran H, . Association between COVID-19 diagnosis and in-hospital mortality in patients hospitalized with ST-segment elevation myocardial infarction. JAMA. 2021;326(19):1940-1952. doi:10.1001/jama.2021.18890 34714327 PMC8596198

[11] de Havenon A, Ney JP, Callaghan B, . Characteristics and outcomes among US patients hospitalized for ischemic stroke before vs during the COVID-19 pandemic. JAMA Netw Open. 2021;4(5):e2110314. doi:10.1001/jamanetworkopen.2021.10314 33999162 PMC8129817

[12] Chinn J, Sedighim S, Kirby KA, . Characteristics and outcomes of women with COVID-19 giving birth at US academic centers during the COVID-19 pandemic. JAMA Netw Open. 2021;4(8):e2120456. doi:10.1001/jamanetworkopen.2021.20456 34379123 PMC8358731

[13] Bilinski A, Emanuel EJ. COVID-19 and excess all-cause mortality in the US and 18 comparison countries. JAMA. 2020;324(20):2100-2102. doi:10.1001/jama.2020.20717 33044514 PMC7551217

[14] 2021 NHSN *ICD-10* operative procedure code mappings. 2021. Accessed August 9, 2021. https://www.cdc.gov/nhsn/xls/guidance-for-hpro-kpro-procedure-details.xlsx

[15] Nuzzo JB, Gostin LO. The first 2 years of COVID-19: lessons to improve preparedness for the next pandemic. JAMA. 2022;327(3):217-218. doi:10.1001/jama.2021.24394 34989763

[16] Sun BC, Hsia RY, Weiss RE, . Effect of emergency department crowding on outcomes of admitted patients. Ann Emerg Med. 2013;61(6):605-611.e6. doi:10.1016/j.annemergmed.2012.10.026 23218508 PMC3690784

[17] Kadri SS, Sun J, Lawandi A, . Association between caseload surge and COVID-19 survival in 558 U.S. hospitals, March to August 2020. Ann Intern Med. 2021;174(9):1240-1251. doi:10.7326/M21-1213 34224257 PMC8276718

[18] CMS releases recommendations on adult elective surgeries, non-essential medical, surgical, and dental procedures during COVID-19 response. Published 2020. Accessed January 10, 2021. https://www.cms.gov/newsroom/press-releases/cms-releases-recommendations-adult-elective-surgeries-non-essential-medical-surgical-and-dental

[19] Mattingly AS, Rose L, Eddington HS, . Trends in US surgical procedures and health care system response to policies curtailing elective surgical operations during the COVID-19 pandemic. JAMA Netw Open. 2021;4(12):e2138038. doi:10.1001/jamanetworkopen.2021.38038 34878546 PMC8655602

[20] Mackey K, Ayers CK, Kondo KK, . Racial and ethnic disparities in COVID-19–related infections, hospitalizations, and deaths: a systematic review. Ann Intern Med. 2021;174(3):362-373. doi:10.7326/M20-6306 33253040 PMC7772883

[21] Rosen H, Saleh F, Lipsitz S, Rogers SO Jr, Gawande AA. Downwardly mobile: the accidental cost of being uninsured. Arch Surg. 2009;144(11):1006-1011. doi:10.1001/archsurg.2009.195 19917936

[22] Baker DW, Sudano JJ, Albert JM, Borawski EA, Dor A. Lack of health insurance and decline in overall health in late middle age. N Engl J Med. 2001;345(15):1106-1112. doi:10.1056/NEJMsa002887 11596591

[23] Memtsoudis SG, Sun X, Chiu YL, . Perioperative comparative effectiveness of anesthetic technique in orthopedic patients. Anesthesiology. 2013;118(5):1046-1058. doi:10.1097/ALN.0b013e318286061d 23612126 PMC3956038

[24] Waits SA, Reames BN, Sheetz KH, Englesbe MJ, Campbell DA Jr. Anticipating the effects of Medicaid expansion on surgical care. JAMA Surg. 2014;149(7):745-747. doi:10.1001/jamasurg.2014.222 24804782 PMC4573587

[25] Torain MJ, Maragh-Bass AC, Dankwa-Mullen I, . Surgical disparities: a comprehensive review and new conceptual framework. J Am Coll Surg. 2016;223(2):408-418. doi:10.1016/j.jamcollsurg.2016.04.047 27296524

[26] Althoff LEF, Ganapati S, Walsh C. The geography of remote work. National Bureau of Economic Research. 2021. Accessed January 11, 2022. https://www.nber.org/papers/w29181

[27] Bundorf MK, Gupta S, Kim C. Trends in health insurance coverage during the COVID-19 pandemic. JAMA Health Forum. 2021;2(9):1-10. doi:10.1001/jamahealthforum.2021.2487 PMC8796896 35977184

[28] Romano PS, Schembri ME, Rainwater JA. Can administrative data be used to ascertain clinically significant postoperative complications? Am J Med Qual. 2002;17(4):145-154. doi:10.1177/106286060201700404 12153067

[29] Lawson EH, Louie R, Zingmond DS, . A comparison of clinical registry versus administrative claims data for reporting of 30-day surgical complications. Ann Surg. 2012;256(6):973-981. doi:10.1097/SLA.0b013e31826b4c4f 23095667

