# Supplementary material for: Association Between the COVID-19 Pandemic and Insurance-Based Disparities in Mortality After Major Surgery Among US Adults
Source: JAMA Netw Open. 2022 Jul 18;5(7):e2222360. doi: 10.1001/jamanetworkopen.2022.22360 (PMC9294995; doi:10.1001/jamanetworkopen.2022.22360)
Supplement: Supplement. — eFigure 1. Flow Diagram Describing Selection of Cases in the Analytic Cohort eFigure 2. Distribution of Hospital COVID-19 Burden eFigure 3. Emergent and Urgent Surgical Case Volumes Stratified by Hospital COVID-19 Burden eFigure 4. Changes in the Proportion of All Patients Undergoing (A) Emergency Surgery and (B) Urgent Surgery Who Have Either Medicaid or No Insurance eTable 1. Patient Characteristics eTable 2. Coefficients for Model Examining the Association Between Changes in All-Cause Inpatient Mortality and Hospital COVID-19 Burden eTable 3. Coefficients for Models Examining the Association Between Changes in All-Cause Inpatient Mortality and Payer Status and Hospital COVID-19 Burden eTable 4. Coefficients for DDD Model Examining the Association Between Changes in All-Cause Inpatient Mortality and Payer Status and Hospital COVID-19 Burden [file jamanetwopen-e2222360-s001.pdf]

Supplementary Online Content

Glance LG, Dick AW, Shippey E, et al. Association between the COVID-19 pandemic and insurance-based disparities in mortality after major surgery among US adults. *JAMA Netw Open*. 2022;5(7):e2222360. doi:10.1001/jamanetworkopen.2022.22360

eFigure 1. Flow Diagram Describing Selection of Cases in the Analytic Cohort
eFigure 2. Distribution of Hospital COVID-19 Burden
eFigure 3. Emergent and Urgent Surgical Case Volumes Stratified by Hospital COVID-19 Burden
eFigure 4. Changes in the Proportion of All Patients Undergoing (A) Emergency Surgery and (B) Urgent Surgery Who Have Either Medicaid or No Insurance
eTable 1. Patient Characteristics
eTable 2. Coefficients for Model Examining the Association Between Changes in All-Cause Inpatient Mortality and Hospital COVID-19 Burden
eTable 3. Coefficients for Models Examining the Association Between Changes in All-Cause Inpatient Mortality and Payer Status and Hospital COVID-19 Burden
eTable 4. Coefficients for DDD Model Examining the Association Between Changes in All-Cause Inpatient Mortality and Payer Status and Hospital COVID-19 Burden

This supplementary material has been provided by the authors to give readers additional information about their work.

© 2022 Glance LG et al. *JAMA Network Open.*

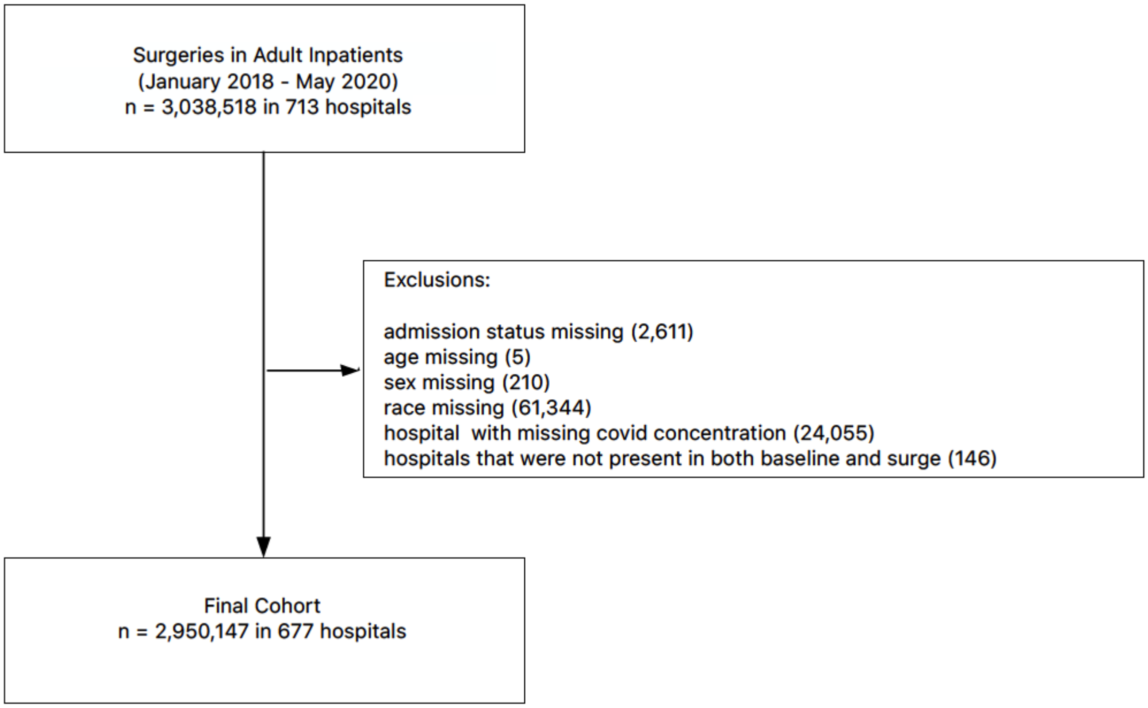

eFigure 1. Flow diagram describing selection of cases in the analytic cohort.

© 2022 Glance LG et al. *JAMA Network Open.*

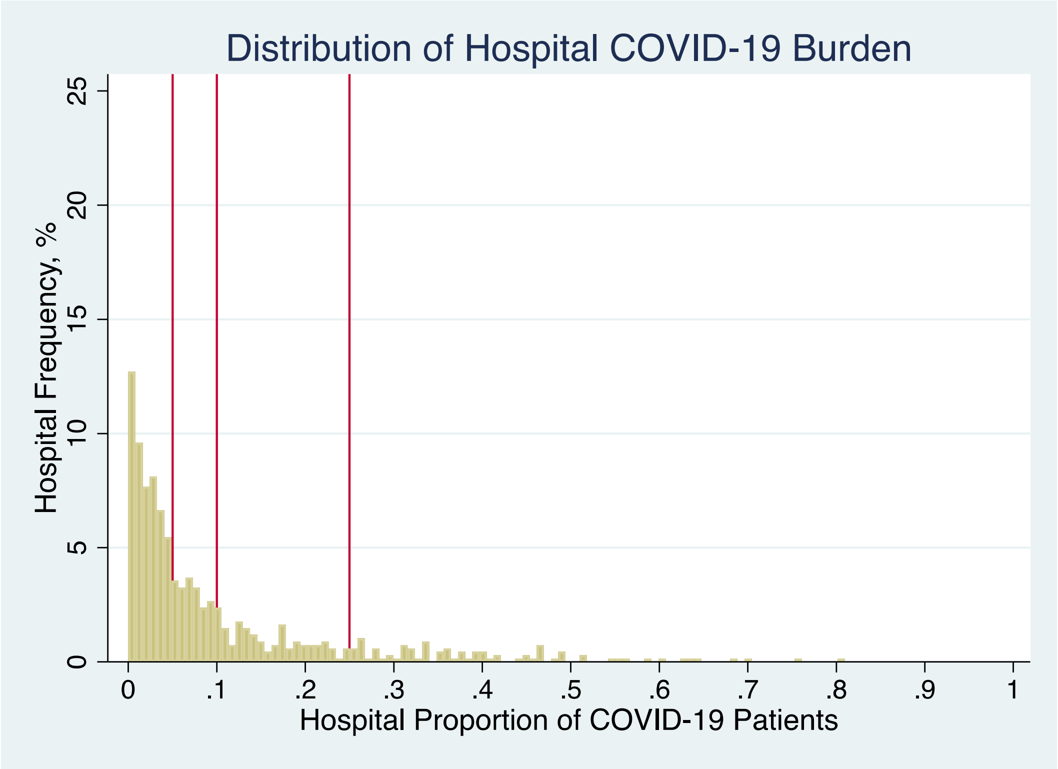

eFigure 2. Distribution of hospital COVID-19 burden.

© 2022 Glance LG et al. *JAMA Network Open.*

A. Emergency surgery case volumes.

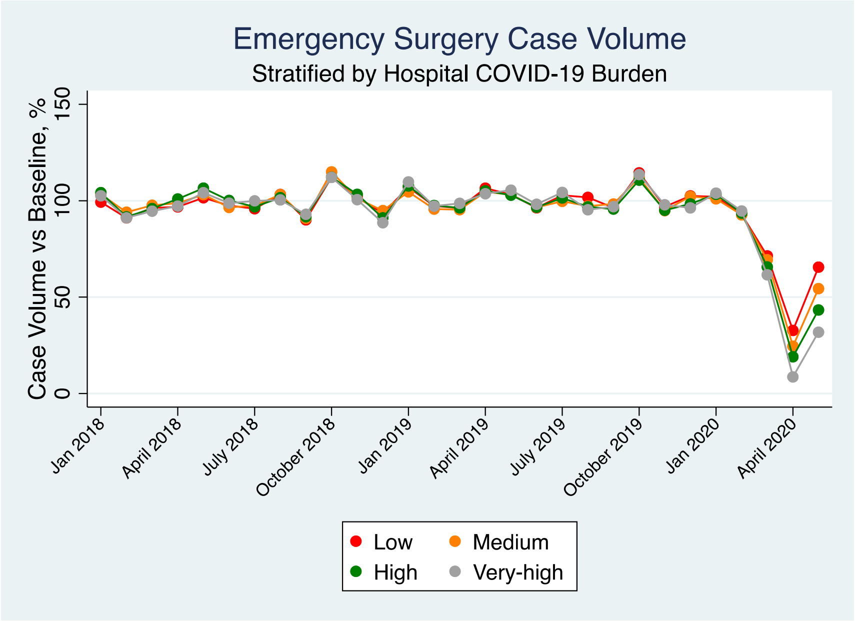

B. Urgent surgery case volumes.

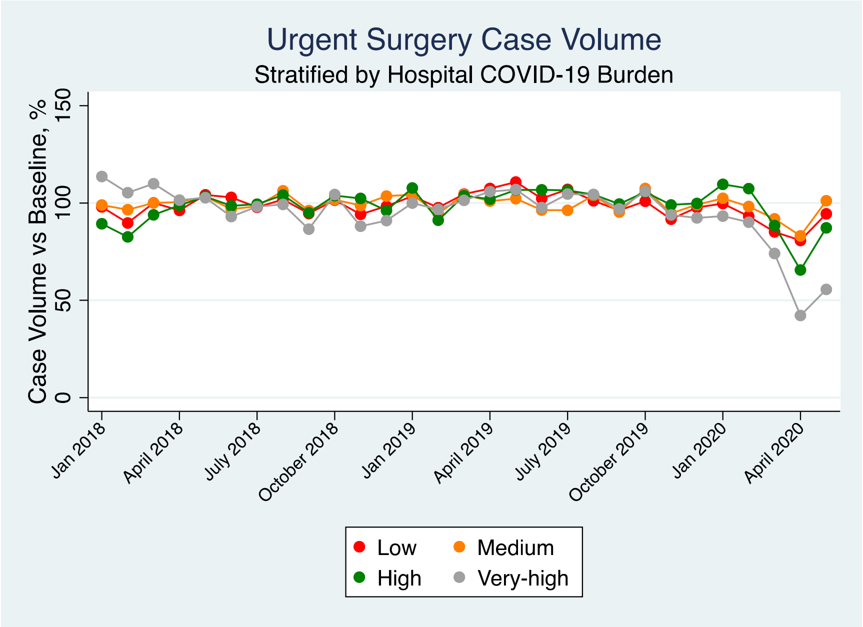

eFigure 3. Emergent and Urgent Surgical Case Volumes Stratified by Hospital COVID-19 burden. The baseline period is between January 1, 2018, to February 29, 2020 and the surge period is between March 1, 2020, and May 31, 2020. Monthly case volumes were normalized by dividing monthly case volumes by mean case volume during the baseline period (January 1, 2018, to February 29, 2020).

© 2022 Glance LG et al. *JAMA Network Open.*

A. Emergency surgery.

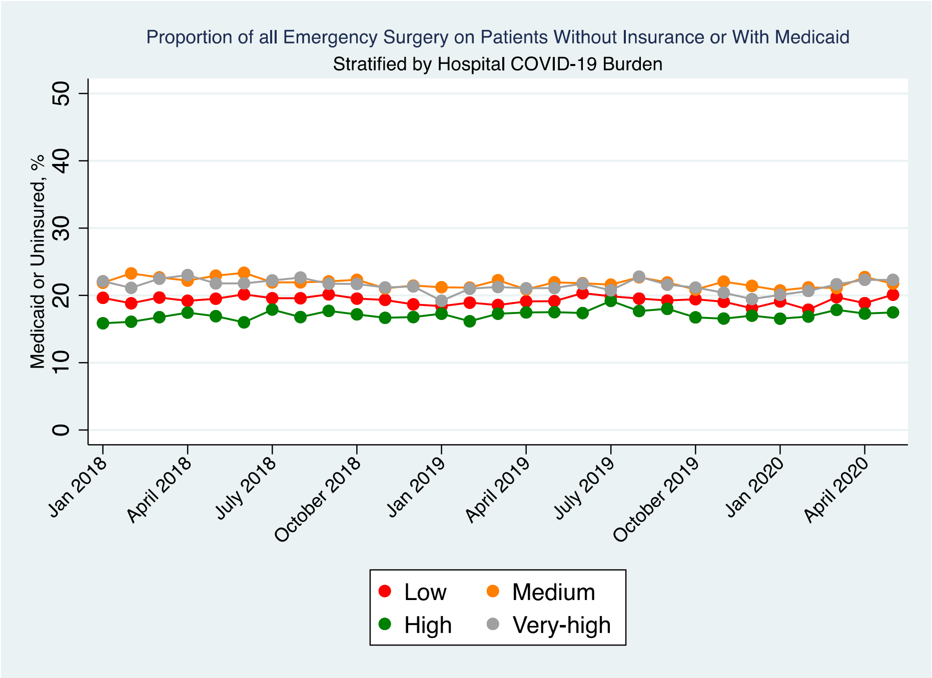

B. Urgent Surgery.

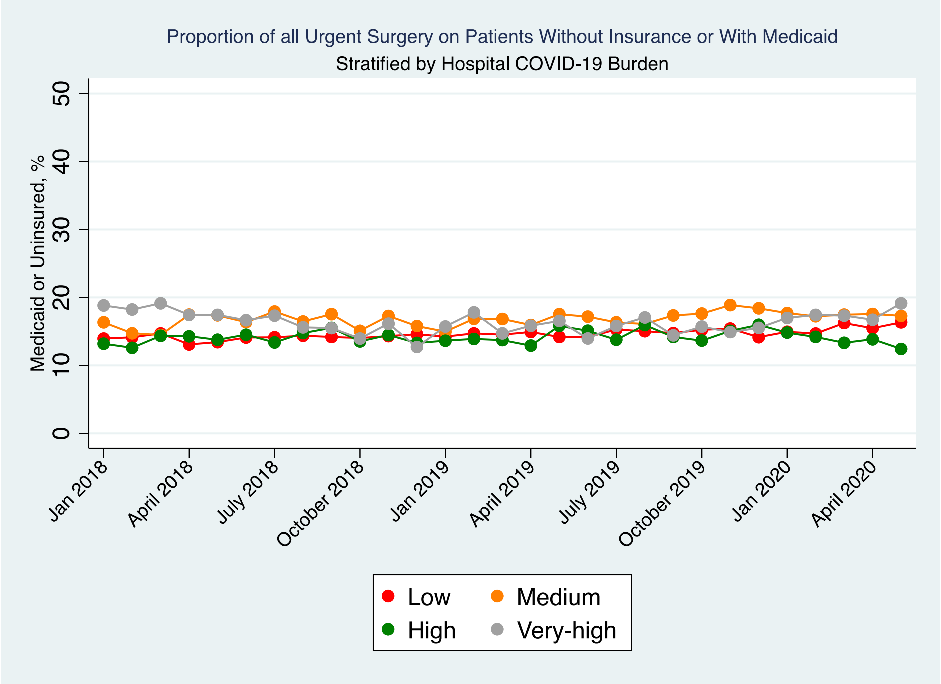

eFigure 4. Changes in the proportion of all patients undergoing (A) emergency surgery and (B) urgent surgery who have either Medicaid or no insurance. The baseline period is between January 1, 2018, to February 29, 2020 and the surge period is between March 1, 2020, and May 31, 2020. Monthly case volumes were normalized by dividing monthly case volumes by mean case volume during the baseline period (January 1, 2018, to February 29, 2020).

© 2022 Glance LG et al. *JAMA Network Open.*

eTable 1. Patient characteristics grouped by payer status.

	Total	Commercial Insurance	Medicare	Medicaid	Uninsured	Other payer
	2,950,147	1,000,068 (33.9)	1,427,791 (48.4)	321,600 (10.9)	59,729 (2.02)	140,959 (4.78)
COVID Surgery Category						
<= 5%	1,414,204 (47.9)	468,163 (46.8)	708,879 (49.7)	141,802 (44.1)	32,403 (54.3)	62,957 (44.7)
5.1-10%	513,795 (17.4)	161,309 (16.1)	243,337 (17)	57,993 (18)	16,520 (27.7)	34,636 (24.6)
10.1-25%	607,068 (20.6)	227,763 (22.8)	286,627 (20.1)	63,070 (19.6)	6,199 (10.4)	23,409 (16.6)
>25%	415,080 (14.1)	142,833 (14.3)	188,948 (13.2)	58,735 (18.3)	4,607 (7.7)	19,957 (14.2)
Time Period						
Baseline	2,747,236 (93.1)	935,033 (93.5)	1,329,865 (93.1)	296,969 (92.3)	54,416 (91.1)	130,953 (92.9)
Surge	202,911 (6.9)	65,035 (6.5)	97,926 (6.9)	24,631 (7.7)	5,313 (8.9)	10,006 (7.1)
Admission status						
Emergent	894,029 (30.3)	232,370 (23.2)	434,120 (30.4)	137,625 (42.8)	38,445 (64.4)	51,469 (36.5)
Urgent	278,239 (9.4)	82,336 (8.2)	141,150 (9.9)	34,280 (10.7)	7,538 (12.6)	12,935 (9.2)
Elective	1,777,879 (60.3)	685,362 (68.5)	852,521 (59.7)	149,695 (46.6)	13,746 (23)	76,555 (54.3)
Sex						
Male	1,399,395 (47.4)	478,146 (47.8)	651,372 (45.6)	146,667 (45.6)	33,508 (56.1)	89,702 (63.6)
Female	1,550,752 (52.6)	521,922 (52.2)	776,419 (54.4)	174,933 (54.4)	26,221 (43.9)	51,257 (36.4)
Age						
18-30	149,731 (5.1)	74,078 (7.4)	4,782 (0.3)	47,672 (14.8)	10,326 (17.3)	12,873 (9.1)
31-50	521,567 (17.7)	282,782 (28.3)	52,258 (3.7)	121,455 (37.8)	24,329 (40.7)	40,743 (28.9)
51-64	918,197 (31.1)	531,128 (53.1)	170,981 (12)	137,770 (42.8)	21,532 (36.1)	56,786 (40.3)
65-74	792,220 (26.9)	93,631 (9.4)	664,564 (46.5)	9,716 (3)	2,321 (3.9)	21,988 (15.6)
75-79	273,891 (9.3)	11,351 (1.1)	254,711 (17.8)	2,537 (0.8)	612 (1)	4,680 (3.3)
80-84	162,233 (5.5)	4,426 (0.4)	153,818 (10.8)	1,475 (0.5)	349 (0.6)	2,165 (1.5)
85-89	86,588 (2.9)	1,879 (0.2)	82,723 (5.8)	664 (0.2)	143 (0.2)	1,179 (0.8)
90 -	45,720 (1.6)	793 (0.1)	43,954 (3.1)	311 (0.1)	117 (0.2)	545 (0.4)
Race						
White	2,299,899 (78)	802,875 (80.3)	1,183,060 (82.9)	182,522 (56.8)	35,171 (58.9)	96,271 (68.3)
Black	381,772 (12.9)	110,739 (11.1)	156,203 (10.9)	78,347 (24.4)	11,926 (20)	24,557 (17.4)
Asian	59,472 (2)	23,072 (2.3)	22,852 (1.6)	9,587 (3)	1,160 (1.9)	2,801 (2)
Other race	209,004 (7.1)	63,382 (6.3)	65,676 (4.6)	51,144 (15.9)	11,472 (19.2)	17,330 (12.3)
Covid-19 positive*	1,969 (0.97)	429 (0.66)	936 (0.96)	440 (1.79)	40 (0.75)	124 (1.24)
Comorbidity						

© 2022 Glance LG et al. *JAMA Network Open.*

Congestive heart failure	201,371 (0.07)	25,601 (0.03)	151,684 (0.11)	16,735 (0.05)	1,840 (0.03)	5,511 (0.04)
Valvular disease	129,786 (0.04)	21,309 (0.02)	97,321 (0.07)	7,212 (0.02)	0,927 (0.02)	3,017 (0.02)
Pulmonary circulation disorder	12,581 (0.004)	3,062 (0.003)	6,185 (0.004)	2,465 (0.01)	0,334 (0.01)	535 (0.004)
Peripheral vascular disease	201,634 (0.07)	37,982 (0.04)	135,150 (0.09)	18,970 (0.06)	2,799 (0.05)	6,733 (0.05)
Hypertension	1,566,188 (0.53)	415,403 (0.42)	934,311 (0.65)	134,430 (0.42)	19,985 (0.33)	62,059 (0.44)
Paralysis	108,107 (0.04)	22,317 (0.02)	62,310 (0.04)	16,902 (0.05)	2,160 (0.04)	4,418 (0.03)
Other neurological disorders	167,790 (0.06)	31,664 (0.03)	110,933 (0.08)	17,877 (0.06)	1,782 (0.03)	5,534 (0.04)
Chronic pulmonary disease	532,640 (0.18)	134,570 (0.13)	304,540 (0.21)	66,159 (0.21)	5,911 (0.1)	21,460 (0.15)
Diabetes, uncomplicated	301,110 (0.1)	92,460 (0.09)	161,774 (0.11)	28,861 (0.09)	3,907 (0.07)	14,108 (0.1)
Diabetes, complicated	419,771 (0.14)	84,541 (0.08)	263,725 (0.18)	46,971 (0.15)	7,747 (0.13)	16,787 (0.12)
Hypothyroidism	407,625 (0.14)	108,680 (0.11)	261,064 (0.18)	23,262 (0.07)	2,674 (0.04)	11,945 (0.08)
Renal failure	344,421 (0.12)	51,425 (0.05)	255,811 (0.18)	24,810 (0.08)	2,716 (0.05)	9,659 (0.07)
Liver disease	141,471 (0.05)	47,067 (0.05)	59,453 (0.04)	25,308 (0.08)	3,421 (0.06)	6,222 (0.04)
Peptic ulcer disease	23,758 (0.01)	5,929 (0.01)	13,134 (0.01)	3,248 (0.01)	0,559 (0.01)	888 (0.01)
AIDS/HIV	5,634 (0.002)	1,142 (0.001)	2,443 (0.002)	1,634 (0.01)	0,116 (0.002)	299 (0.002)
Lymphoma	16,384 (0.01)	3,928 (0.004)	10,863 (0.01)	1,009 (0)	0,114 (0.002)	470 (0.003)
Metastatic cancer	114,544 (0.04)	42,396 (0.04)	55,295 (0.04)	12,122 (0.04)	1,410 (0.02)	3,321 (0.02)
Solid tumor without metastasis	64,960 (0.02)	19,077 (0.02)	36,882 (0.03)	6,100 (0.02)	0,704 (0.01)	2,197 (0.02)
Rheumatoid arthritis	114,375 (0.04)	29,852 (0.03)	71,819 (0.05)	8,529 (0.03)	0,751 (0.01)	3,424 (0.02)
Coagulopathy	108,160 (0.04)	27,099 (0.03)	61,083 (0.04)	13,653 (0.04)	1,980 (0.03)	4,345 (0.03)
Obesity	681,014 (0.23)	246,911 (0.25)	316,807 (0.22)	74,563 (0.23)	11,198 (0.19)	31,535 (0.22)
Weight loss	172,439 (0.06)	39,583 (0.04)	99,610 (0.07)	24,615 (0.08)	2,902 (0.05)	5,729 (0.04)
Fluid & electrolyte disorder	360,323 (0.12)	83,469 (0.08)	202,047 (0.14)	50,019 (0.16)	10,473 (0.18)	14,315 (0.1)
Blood loss anemia	22,258 (0.01)	6,127 (0.01)	10,616 (0.01)	4,259 (0.01)	0,480 (0.01)	776 (0.01)
Deficiency anemia	356,635 (0.12)	80,882 (0.08)	211,117 (0.15)	45,839 (0.14)	6,368 (0.11)	12,429 (0.09)
Alcohol abuse	80,421 (0.03)	20,467 (0.02)	30,762 (0.02)	19,987 (0.06)	4,075 (0.07)	5,130 (0.04)
Drug abuse	60,372 (0.02)	10,140 (0.01)	18,768 (0.01)	24,454 (0.08)	3,302 (0.06)	3,708 (0.03)
Psychoses	83,291 (0.03)	15,794 (0.02)	43,024 (0.03)	19,267 (0.06)	1,333 (0.02)	3,873 (0.03)
Depression	423,122 (0.14)	126,554 (0.13)	222,569 (0.16)	51,394 (0.16)	4,628 (0.08)	17,977 (0.13)
Procedure						
AAA repair	3,621 (0.12)	956 (0.1)	2,105 (0.15)	351 (0.11)	73 (0.12)	136 (0.1)
Amputation	132,707 (4.5)	22,555 (2.26)	74,016 (5.18)	24,250 (7.54)	4,888 (8.18)	6,998 (4.96)
Appendectomy	78,680 (2.67)	37,697 (3.77)	19,042 (1.33)	12,127 (3.77)	5,445 (9.12)	4,369 (3.1)
Bile duct, liver or pancreas	87,594 (2.97)	36,015 (3.6)	36,238 (2.54)	10,388 (3.23)	1,535 (2.57)	3,418 (2.42)
Cardiac surgery	131,796 (4.47)	46,700 (4.67)	65,208 (4.57)	13,327 (4.14)	2,010 (3.37)	4,551 (3.23)

© 2022 Glance LG et al. *JAMA Network Open.*

CABG	101,909 (3.45)	32,429 (3.24)	54,850 (3.84)	8,259 (2.57)	2,338 (3.91)	4,033 (2.86)
Carotid endarterectomy	37,283 (1.26)	6,423 (0.64)	27,730 (1.94)	1,810 (0.56)	384 (0.64)	0,936 (0.66)
Cholecystectomy	155,052 (5.26)	53,448 (5.34)	57,448 (4.02)	26,826 (8.34)	9,786 (16.38)	7,544 (5.35)
Colon surgery	208,139 (7.06)	79,125 (7.91)	96,176 (6.74)	22,051 (6.86)	3,856 (6.46)	6,931 (4.92)
Craniotomy	230,533 (7.81)	86,008 (8.6)	95,486 (6.69)	32,648 (10.15)	5,524 (9.25)	10,867 (7.71)
Spinal fusion	280,584 (9.51)	95,452 (9.54)	130,315 (9.13)	26,726 (8.31)	2,455 (4.11)	25,636 (18.2)
Fracture surgery	178,368 (6.05)	36,184 (3.62)	98,266 (6.88)	20,038 (6.23)	6,725 (11.26)	17,155 (12.2)
Gastric surgery	137,453 (4.66)	69,100 (6.91)	39,892 (2.79)	21,615 (6.72)	2,244 (3.76)	4,602 (3.26)
Hip arthroplasty	331,585 (11.24)	98,959 (9.9)	202,792 (14.2)	18,453 (5.74)	1,714 (2.87)	9,667 (6.86)
Knee arthroplasty	345,283 (11.7)	115,620 (11.6)	198,997 (13.9)	17,004 (5.29)	922 (1.54)	12,740 (9.04)
Laminectomy	68,394 (2.32)	22,018 (2.2)	34,212 (2.4)	6,599 (2.05)	902 (1.51)	4,663 (3.31)
Peripheral vascular bypass	38,647 (1.31)	8,065 (0.81)	23,607 (1.65)	4,860 (1.51)	685 (1.15)	1,430 (1.01)
Small bowel surgery	122,676 (4.16)	53,544 (5.35)	45,232 (3.17)	17,216 (5.35)	2,167 (3.63)	4,517 (3.2)
Thoracic surgery	131,991 (4.47)	44,292 (4.43)	66,068 (4.63)	14,166 (4.4)	2,586 (4.33)	4,879 (3.46)
Exploratory laparotomy	147,852 (5.01)	55,478 (5.55)	60,111 (4.21)	22,886 (7.12)	3,490 (5.84)	5,887 (4.18)
Death	50,816 (1.72)	9,158 (0.92)	31,943 (2.24)	6,142 (1.91)	1,416 (2.37)	2,157 (1.53)
* percentage of patients who tested COVID-19 positive during the Surge Period						

© 2022 Glance LG et al. *JAMA Network Open.*

**eTable 2.** Model examining the association between changes in all-cause inpatient mortality and hospital COVID-19 burden.								
	All Surgery		Elective Surgery		Urgent Surgery		Emergent Surgery	
	AOR (95% CI)	P	AOR (95% CI)	P	AOR (95% CI)	P	AOR (95% CI)	P
Hospital COVID Burden								
<= 5%	reference		reference		reference		reference	
5.1-10.0%	0.99 (0.88,1.12)	0.923	1.03 (0.87,1.22)	0.752	1.16 (0.99,1.35)	0.061	0.94 (0.83,1.07)	0.370
10.1-25.0%	0.94 (0.86,1.03)	0.215	0.88 (0.76,1.02)	0.091	1.12 (0.96,1.3)	0.141	0.92 (0.84,1.01)	0.083
>25%	0.88 (0.78,0.99)	0.040	0.84 (0.69,1.01)	0.059	1.2 (0.99,1.44)	0.057	0.85 (0.76,0.96)	0.007
Time Period								
Baseline	reference		reference		reference		reference	
Surge	0.9 (0.85,0.94)	<.001	1 (0.87,1.14)	0.956	0.83 (0.75,0.93)	0.001	0.89 (0.84,0.95)	<.001
Hospital COVID burden X Surge								
<=5% X Surge	reference		reference		reference		reference	
5.1-10% X Surge	1.05 (0.96,1.15)	0.299	1.02 (0.77,1.33)	0.909	1.07 (0.86,1.34)	0.531	1.06 (0.95,1.18)	0.316
10.1-25% X Surge	1.13 (1.03,1.24)	0.013	1.27 (1.01,1.6)	0.044	1.05 (0.8,1.37)	0.739	1.1 (1,1.22)	0.056
>25% X Surge	1.38 (1.24,1.53)	<.001	0.94 (0.66,1.33)	0.719	1.59 (1.27,2)	<.001	1.4 (1.24,1.59)	<.001
Admission status								
Emergent	4.68 (4.4,4.98)	<.001						
Urgent	4.03 (3.73,4.36)	<.001						
Elective	reference							
Race								
White	reference		reference		reference		reference	
Black	1.02 (0.97,1.07)	0.466	1.15 (1.04,1.26)	0.006	0.99 (0.91,1.08)	0.809	1 (0.95,1.06)	0.865
Asian	1 (0.93,1.08)	0.966	1.32 (1.13,1.55)	<.001	1.16 (1,1.35)	0.047	0.91 (0.84,1)	0.052
Other race	1.07 (1,1.14)	0.045	1.15 (1.02,1.29)	0.019	1.18 (1.07,1.3)	0.001	1.02 (0.95,1.1)	0.556
Payer								
Commercial insurance	reference		reference		reference		reference	
Medicare	1.27 (1.21,1.33)	<.001	1.38 (1.26,1.52)	<.001	1.18 (1.07,1.29)	<.001	1.22 (1.16,1.29)	<.001
Medicaid	1.29 (1.23,1.36)	<.001	1.4 (1.24,1.58)	<.001	1.23 (1.12,1.36)	<.001	1.23 (1.16,1.3)	<.001
Uninsured	1.73 (1.54,1.94)	<.001	2.03 (1.53,2.69)	<.001	1.49 (1.23,1.79)	<.001	1.61 (1.43,1.83)	<.001
Other payer	1.43 (1.32,1.55)	<.001	1.58 (1.36,1.83)	<.001	1.1 (0.95,1.28)	0.215	1.43 (1.31,1.57)	<.001

© 2022 Glance LG et al. *JAMA Network Open.*

Sex								
Male	reference		reference		reference		reference	
Female	0.93 (0.91,0.96)	<.001	0.9 (0.85,0.96)	0.001	0.98 (0.93,1.03)	0.340	0.95 (0.92,0.98)	0.001
Age								
18-30	reference		reference		reference		reference	
31-50	1.13 (1.05,1.21)	0.001	1.21 (0.96,1.53)	0.105	1.29 (1.13,1.47)	<.001	1.12 (1.04,1.22)	0.004
51-64	1.49 (1.38,1.61)	<.001	2.07 (1.66,2.59)	<.001	1.61 (1.41,1.84)	<.001	1.43 (1.32,1.56)	<.001
65-74	1.82 (1.68,1.97)	<.001	2.57 (2.04,3.23)	<.001	1.96 (1.69,2.28)	<.001	1.73 (1.59,1.88)	<.001
75-79	2.16 (1.98,2.35)	<.001	3.34 (2.62,4.25)	<.001	2.23 (1.91,2.62)	<.001	1.97 (1.8,2.16)	<.001
80-84	2.4 (2.2,2.61)	<.001	4.21 (3.29,5.4)	<.001	2.49 (2.09,2.95)	<.001	2.12 (1.93,2.33)	<.001
85-89	2.7 (2.46,2.95)	<.001	4.97 (3.85,6.43)	<.001	2.5 (2.07,3.02)	<.001	2.46 (2.22,2.72)	<.001
90 -	3.39 (3.09,3.73)	<.001	7.24 (5.38,9.73)	<.001	3.03 (2.44,3.75)	<.001	3.1 (2.81,3.43)	<.001
Covid positive	5.55 (4.75,6.49)	<.001	22.23 (11.44,43.2)	<.001	5.23 (3.7,7.41)	<.001	5.23 (4.44,6.17)	<.001
Comorbidity								
Congestive heart failure	1.88 (1.82,1.94)	<.001	2.78 (2.54,3.04)	<.001	2 (1.88,2.13)	<.001	1.74 (1.67,1.8)	<.001
Valvular disease	1.29 (1.23,1.34)	<.001	1.97 (1.78,2.18)	<.001	1.34 (1.23,1.46)	<.001	1.19 (1.14,1.24)	<.001
Pulmonary circulation disorder	1.57 (1.45,1.7)	<.001	3.49 (2.76,4.4)	<.001	1.6 (1.38,1.86)	<.001	1.48 (1.35,1.63)	<.001
Peripheral vascular disease	1.73 (1.67,1.78)	<.001	1.92 (1.78,2.08)	<.001	1.7 (1.6,1.8)	<.001	1.67 (1.61,1.73)	<.001
Hypertension	1.01 (0.98,1.05)	0.473	1.2 (1.1,1.3)	<.001	1 (0.93,1.07)	0.972	0.98 (0.94,1.01)	0.162
Paralysis	1.19 (1.14,1.25)	<.001	2.05 (1.81,2.33)	<.001	1.28 (1.17,1.4)	<.001	1.07 (1.01,1.12)	0.014
Other neurological disorders	1.13 (1.09,1.17)	<.001	1.26 (1.14,1.39)	<.001	1.09 (1,1.18)	0.048	1.09 (1.04,1.14)	<.001
Chronic pulmonary disease	1.17 (1.14,1.21)	<.001	1.19 (1.12,1.27)	<.001	1.16 (1.1,1.23)	<.001	1.17 (1.13,1.21)	<.001
Diabetes, uncomplicated	0.96 (0.92,1)	0.043	0.85 (0.77,0.94)	0.002	0.94 (0.87,1.02)	0.126	1.01 (0.96,1.06)	0.683
Diabetes, complicated	1.07 (1.04,1.1)	<.001	1.1 (1.02,1.18)	0.009	1.05 (1,1.11)	0.070	1.06 (1.02,1.1)	0.001
Hypothyroidism	0.98 (0.96,1.01)	0.155	1.05 (0.99,1.12)	0.104	1.01 (0.95,1.07)	0.747	0.95 (0.92,0.99)	0.004
Renal failure	1.61 (1.56,1.66)	<.001	1.95 (1.83,2.08)	<.001	1.61 (1.53,1.7)	<.001	1.54 (1.48,1.59)	<.001
Liver disease	1.43 (1.38,1.49)	<.001	1.66 (1.51,1.81)	<.001	1.51 (1.39,1.63)	<.001	1.39 (1.33,1.45)	<.001
Peptic ulcer disease	1.43 (1.33,1.52)	<.001	1.61 (1.32,1.97)	<.001	1.41 (1.22,1.64)	<.001	1.4 (1.3,1.51)	<.001
AIDS/HIV	1.04 (0.86,1.26)	0.659	1.11 (0.66,1.88)	0.689	0.88 (0.51,1.51)	0.635	1.03 (0.83,1.28)	0.762
Lymphoma	1.36 (1.24,1.48)	<.001	1.36 (1.05,1.74)	0.018	1.46 (1.21,1.75)	<.001	1.3 (1.17,1.45)	<.001
Metastatic cancer	1.45 (1.39,1.52)	<.001	1.65 (1.49,1.83)	<.001	1.32 (1.21,1.45)	<.001	1.38 (1.31,1.45)	<.001
Solid tumor without metastasis	0.93 (0.88,0.98)	0.010	1.12 (0.98,1.29)	0.108	0.89 (0.79,1)	0.044	0.9 (0.84,0.96)	0.002
Rheumatoid arthritis	1.14 (1.08,1.19)	<.001	1.2 (1.07,1.34)	0.002	1.06 (0.95,1.19)	0.312	1.13 (1.07,1.19)	<.001
Coagulopathy	2.53 (2.42,2.64)	<.001	2.07 (1.88,2.27)	<.001	2.31 (2.16,2.46)	<.001	2.63 (2.51,2.77)	<.001
Obesity	1.07 (1.04,1.11)	<.001	1.25 (1.17,1.35)	<.001	1.1 (1.04,1.18)	0.002	1.01 (0.98,1.05)	0.466

© 2022 Glance LG et al. *JAMA Network Open.*

Weight loss	1.4 (1.34,1.46)	<.001	2.63 (2.43,2.86)	<.001	1.35 (1.25,1.45)	<.001	1.28 (1.22,1.33)	<.001
Fluid & electrolyte disorder	2.3 (2.23,2.37)	<.001	2.23 (2.07,2.42)	<.001	2.42 (2.3,2.55)	<.001	2.24 (2.16,2.31)	<.001
Blood loss anemia	0.8 (0.73,0.88)	<.001	0.93 (0.72,1.21)	0.607	0.88 (0.73,1.06)	0.184	0.75 (0.68,0.84)	<.001
Deficiency anemia	0.95 (0.92,0.98)	0.004	1.25 (1.15,1.35)	<.001	1.02 (0.96,1.09)	0.522	0.9 (0.87,0.93)	<.001
Alcohol abuse	1.17 (1.12,1.23)	<.001	1.55 (1.36,1.77)	<.001	1.12 (1.01,1.25)	0.029	1.11 (1.05,1.16)	<.001
Drug abuse	0.88 (0.81,0.95)	0.001	1.06 (0.85,1.32)	0.591	0.84 (0.73,0.96)	0.012	0.87 (0.8,0.96)	0.003
Psychoses	0.89 (0.84,0.94)	<.001	1.09 (0.92,1.29)	0.303	0.89 (0.77,1.01)	0.077	0.85 (0.8,0.91)	<.001
Depression	0.84 (0.81,0.87)	<.001	0.95 (0.88,1.03)	0.217	0.85 (0.8,0.91)	<.001	0.82 (0.79,0.85)	<.001
Procedure								
AAA repair	18.57 (15.76,21.89)	<.001	6.22 (4.66,8.31)	<.001	12.18 (8.68,17.08)	<.001	19.81 (15.86,24.75)	<.001
Amputation	1.65 (1.52,1.8)	<.001	0.57 (0.44,0.74)	<.001	1.48 (1.25,1.75)	<.001	1.97 (1.8,2.17)	<.001
Appendectomy	1.32 (1.16,1.5)	<.001	0.8 (0.58,1.1)	0.176	1.56 (1.17,2.08)	0.003	1.27 (1.09,1.46)	0.002
Bile duct, liver or pancreas	5.42 (4.87,6.03)	<.001	1.59 (1.25,2.01)	<.001	3.54 (2.88,4.36)	<.001	5.79 (5.1,6.58)	<.001
Cardiac surgery	10.8 (9.73,11.98)	<.001	4.02 (3.14,5.16)	<.001	7.35 (6.17,8.75)	<.001	11.47 (10.22,12.88)	<.001
CABG	2.93 (2.62,3.28)	<.001	1.44 (1.12,1.86)	0.005	1.88 (1.5,2.35)	<.001	2.97 (2.6,3.38)	<.001
CABG with IMA (no vein)	3.51 (2.85,4.33)	<.001	1.37 (0.87,2.15)	0.177	2.21 (1.42,3.45)	<.001	3.77 (2.75,5.16)	<.001
Carotid endarterectomy	0.99 (0.81,1.2)	0.893	0.22 (0.15,0.32)	<.001	0.88 (0.54,1.45)	0.622	1.06 (0.8,1.39)	0.691
Cholecystectomy	reference		reference		reference		reference	
Colon surgery	5.72 (5.29,6.19)	<.001	0.89 (0.71,1.12)	0.313	4.82 (4.1,5.67)	<.001	7.67 (7.02,8.38)	<.001
Craniotomy	12.06 (10.91,13.34)	<.001	1.44 (1.13,1.85)	0.004	9.09 (7.71,10.73)	<.001	16.36 (14.65,18.26)	<.001
Spinal fusion	1.62 (1.45,1.82)	<.001	0.24 (0.18,0.31)	<.001	1.48 (1.17,1.88)	0.001	2.65 (2.34,3.01)	<.001
Fracture surgery	0.93 (0.84,1.02)	0.132	0.2 (0.13,0.31)	<.001	0.87 (0.73,1.04)	0.138	1.13 (1.02,1.27)	0.024
Gastric surgery	4.57 (4.15,5.03)	<.001	0.88 (0.69,1.13)	0.320	3.96 (3.22,4.87)	<.001	6.4 (5.72,7.16)	<.001
Hip arthroplasty	0.92 (0.83,1.02)	0.122	0.1 (0.07,0.13)	<.001	1.13 (0.91,1.41)	0.276	1.32 (1.18,1.47)	<.001
Knee arthroplasty	0.4 (0.34,0.47)	<.001	0.05 (0.03,0.06)	<.001	0.59 (0.41,0.86)	0.005	1.18 (0.94,1.5)	0.159
Laminectomy	1.38 (1.21,1.58)	<.001	0.19 (0.13,0.26)	<.001	1.24 (0.94,1.63)	0.133	1.94 (1.64,2.29)	<.001
Peripheral vascular bypass	4.18 (3.74,4.67)	<.001	1.31 (0.99,1.72)	0.057	3.03 (2.41,3.81)	<.001	4.58 (3.99,5.27)	<.001
Small bowel surgery	4.89 (4.48,5.33)	<.001	0.91 (0.72,1.17)	0.470	4.92 (4.1,5.91)	<.001	6.12 (5.54,6.76)	<.001
Thoracic surgery	5.58 (5.1,6.11)	<.001	0.96 (0.76,1.21)	0.746	5.36 (4.52,6.36)	<.001	7.12 (6.44,7.88)	<.001
Exploratory laparotomy	4.49 (4.15,4.86)	<.001	0.97 (0.77,1.24)	0.833	3.77 (3.16,4.5)	<.001	5.54 (5.07,6.04)	<.001

© 2022 Glance LG et al. *JAMA Network Open.*

eTable 3. Coefficients for models examining the association between changes in all-cause inpatient mortality and payer status & hospital COVID-19 burden

	All Hospitals		Hospital COVID19 <= 5%		Hospital COVID19 5.1-10%		Hospital COVID19 10.1-25%		Hospital COVID19 >25 %	
	AOR (95% CI)	P	AOR (95% CI)	P	AOR (95% CI)	P	AOR (95% CI)	P	AOR (95% CI)	P
Payer										
Commercial insurance	reference		reference		reference		reference		reference	
Medicare	1.28 (1.22,1.34)	<.001	1.22 (1.12,1.32)	<.001	1.38 (1.26,1.51)	<.001	1.27 (1.17,1.37)	<.001	1.35 (1.21,1.5)	<.001
Medicaid	1.29 (1.22,1.36)	<.001	1.27 (1.17,1.37)	<.001	1.4 (1.27,1.55)	<.001	1.21 (1.08,1.35)	<.001	1.35 (1.19,1.53)	<.001
Uninsured	1.75 (1.55,1.98)	<.001	1.49 (1.28,1.75)	<.001	1.81 (1.45,2.27)	<.001	2.42 (1.91,3.07)	<.001	2.25 (1.4,3.6)	0.001
Other payer	1.42 (1.31,1.54)	<.001	1.31 (1.16,1.47)	<.001	1.39 (1.13,1.69)	0.001	1.62 (1.42,1.85)	0.001	1.64 (1.4,1.92)	<.001
Time Period										
Baseline	reference		reference		reference		reference		reference	
Surge	0.97 (0.91,1.05)	0.477	0.91 (0.82,1.02)	0.105	0.88 (0.77,1)	0.056	1.11 (0.96,1.28)	0.169	1.15 (0.95,1.4)	0.158
Payer X Surge										
Commercial insurance X Surge										
Medicare X Surge	0.95 (0.88,1.03)	0.233	0.94 (0.83,1.07)	0.367	1.06 (0.89,1.26)	0.504	0.88 (0.75,1.02)	0.098	1.01 (0.81,1.25)	0.951
Medicaid X Surge	1.08 (0.96,1.2)	0.204	1.03 (0.85,1.24)	0.778	1.19 (0.96,1.48)	0.118	1.03 (0.82,1.3)	0.790	1.13 (0.86,1.48)	0.378
Uninsured X Surge	1.09 (0.9,1.32)	0.381	1.23 (0.93,1.62)	0.147	1.1 (0.76,1.58)	0.619	0.85 (0.47,1.54)	0.601	1.28 (0.65,2.52)	0.468
Other payer X Surge	1.17 (1.01,1.36)	0.040	1.2 (0.96,1.52)	0.114	1.29 (0.99,1.68)	0.059	1.19 (0.85,1.67)	0.303	1.07 (0.65,1.77)	0.775
Admission status										
Emergent	4.67 (4.39,4.98)	<.001	4.79 (4.34,5.28)	<.001	4.36 (3.77,5.05)	<.001	4.84 (4.38,5.33)	<.001	4.54 (3.88,5.31)	<.001
Urgent	4.06 (3.76,4.38)	<.001	3.73 (3.3,4.21)	<.001	4.09 (3.54,4.72)	<.001	4.49 (3.9,5.16)	<.001	5.12 (4.25,6.18)	<.001
Elective	reference		reference		reference		reference		reference	
Sex										
Male	reference		reference		reference		reference		reference	
Female	0.93 (0.91,0.96)	<.001	0.96 (0.92,0.99)	0.015	0.94 (0.89,1)	0.039	0.94 (0.89,1)	0.035	0.85 (0.81,0.9)	<.001
Age										
18-30	reference		reference		reference		reference		reference	
31-50	1.12 (1.05,1.21)	0.002	1.12 (1.02,1.22)	0.015	1.08 (0.89,1.32)	0.428	1.22 (1.08,1.37)	0.001	1.07 (0.85,1.35)	0.566

© 2022 Glance LG et al. *JAMA Network Open.*

51-64	1.48 (1.37,1.6)	<.001	1.51 (1.37,1.66)	<.001	1.46 (1.15,1.85)	0.002	1.53 (1.35,1.72)	<.001	1.39 (1.16,1.68)	0.001
65-74	1.8 (1.66,1.95)	<.001	1.88 (1.7,2.09)	<.001	1.7 (1.35,2.13)	<.001	1.9 (1.64,2.19)	<.001	1.62 (1.3,2.01)	<.001
75-79	2.13 (1.95,2.32)	<.001	2.24 (2,2.5)	<.001	2 (1.55,2.57)	<.001	2.2 (1.9,2.54)	<.001	1.97 (1.59,2.44)	<.001
80-84	2.36 (2.17,2.57)	<.001	2.47 (2.21,2.76)	<.001	2.22 (1.73,2.84)	<.001	2.49 (2.16,2.87)	<.001	2.16 (1.7,2.73)	<.001
85-89	2.65 (2.42,2.92)	<.001	2.77 (2.43,3.16)	<.001	2.71 (2.08,3.52)	<.001	2.49 (2.14,2.9)	<.001	2.58 (2.06,3.23)	<.001
90 -	3.34 (3.03,3.68)	<.001	3.39 (2.95,3.89)	<.001	2.86 (2.22,3.69)	<.001	3.59 (3,4.29)	<.001	3.6 (2.87,4.52)	<.001
Covid positive	5.98 (5.11,7)	<.001	5.19 (3.25,8.29)	<.001	5.26 (3.44,8.05)	<.001	4.36 (3.21,5.92)	<.001	6.62 (5.43,8.06)	<.001
Comorbidity										
Congestive heart failure	1.88 (1.82,1.94)	<.001	1.87 (1.77,1.97)	<.001	1.84 (1.71,1.98)	<.001	1.94 (1.81,2.08)	<.001	1.9 (1.74,2.08)	<.001
Valvular disease	1.28 (1.23,1.34)	<.001	1.31 (1.23,1.39)	<.001	1.29 (1.17,1.43)	<.001	1.24 (1.14,1.36)	<.001	1.26 (1.14,1.4)	<.001
Pulmonary circulation disorder	1.57 (1.45,1.7)	<.001	1.6 (1.43,1.79)	<.001	1.69 (1.36,2.1)	<.001	1.43 (1.22,1.66)	<.001	1.56 (1.28,1.89)	<.001
Peripheral vascular disease	1.73 (1.67,1.78)	<.001	1.8 (1.72,1.88)	<.001	1.73 (1.61,1.87)	<.001	1.71 (1.6,1.82)	<.001	1.51 (1.39,1.63)	<.001
Hypertension	1.01 (0.98,1.05)	0.419	1.04 (0.99,1.1)	<.001	1 (0.93,1.07)	<.001	0.98 (0.92,1.04)	0.507	0.98 (0.9,1.07)	0.686
Paralysis	1.19 (1.14,1.25)	<.001	1.19 (1.11,1.28)	0.112	1.1 (0.98,1.24)	0.971	1.3 (1.18,1.42)	<.001	1.18 (1.05,1.31)	0.005
Other neurological disorders	1.13 (1.09,1.17)	<.001	1.14 (1.08,1.21)	<.001	1.07 (0.99,1.15)	0.094	1.08 (0.99,1.17)	0.096	1.23 (1.13,1.34)	<.001
Chronic pulmonary disease	1.17 (1.13,1.21)	<.001	1.18 (1.14,1.23)	<.001	1.16 (1.06,1.27)	0.104	1.17 (1.1,1.24)	<.001	1.13 (1.03,1.23)	0.007
Diabetes, uncomplicated	0.96 (0.92,1)	0.044	0.94 (0.89,0.99)	<.001	0.99 (0.89,1.1)	0.001	0.98 (0.9,1.08)	0.740	0.97 (0.88,1.07)	0.560
Diabetes, complicated	1.07 (1.04,1.1)	<.001	1.08 (1.03,1.12)	0.031	1.06 (0.99,1.14)	0.832	1.07 (1.02,1.13)	0.007	1.08 (0.99,1.18)	0.095
Hypothyroidism	0.98 (0.96,1.01)	0.153	0.99 (0.95,1.02)	0.001	0.95 (0.9,1.01)	0.096	1 (0.94,1.06)	0.875	0.96 (0.9,1.03)	0.269
Renal failure	1.61 (1.56,1.66)	<.001	1.58 (1.52,1.65)	0.456	1.49 (1.39,1.6)	0.137	1.71 (1.61,1.82)	<.001	1.74 (1.61,1.89)	<.001
Liver disease	1.44 (1.38,1.49)	<.001	1.4 (1.32,1.49)	<.001	1.47 (1.34,1.62)	<.001	1.49 (1.39,1.6)	<.001	1.41 (1.25,1.59)	<.001
Peptic ulcer disease	1.43 (1.33,1.52)	<.001	1.4 (1.27,1.55)	<.001	1.44 (1.25,1.66)	<.001	1.53 (1.32,1.77)	<.001	1.34 (1.12,1.6)	0.001
AIDS/HIV	1.05 (0.87,1.27)	0.636	1.18 (0.89,1.56)	<.001	1.04 (0.72,1.5)	<.001	0.73 (0.41,1.32)	0.305	1.18 (0.78,1.79)	0.435
Lymphoma	1.35 (1.24,1.48)	<.001	1.35 (1.18,1.54)	0.256	1.7 (1.36,2.14)	0.815	1.2 (0.99,1.44)	0.057	1.23 (1,1.52)	0.049
Metastatic cancer	1.45 (1.39,1.51)	<.001	1.41 (1.32,1.5)	<.001	1.45 (1.32,1.61)	<.001	1.42 (1.3,1.55)	<.001	1.69 (1.5,1.9)	<.001
Solid tumor without metastasis	0.93 (0.88,0.98)	0.009	0.94 (0.87,1.01)	<.001	0.93 (0.83,1.05)	<.001	0.88 (0.76,1.02)	0.096	1 (0.87,1.13)	0.956
Rheumatoid arthritis	1.14 (1.08,1.19)	<.001	1.15 (1.07,1.23)	0.072	1.06 (0.94,1.19)	0.245	1.11 (0.99,1.24)	0.077	1.26 (1.12,1.41)	<.001
Coagulopathy	2.53 (2.42,2.64)	<.001	2.51 (2.36,2.68)	<.001	2.47 (2.22,2.74)	0.329	2.61 (2.41,2.82)	<.001	2.58 (2.33,2.85)	<.001
Obesity	1.07 (1.03,1.11)	<.001	1.08 (1.03,1.12)	<.001	1.08 (0.98,1.2)	<.001	1.05 (0.97,1.14)	0.205	1.08 (0.99,1.16)	0.070
Weight loss	1.4 (1.34,1.46)	<.001	1.38 (1.29,1.47)	0.001	1.3 (1.18,1.44)	0.130	1.46 (1.33,1.6)	<.001	1.52 (1.39,1.67)	<.001
Fluid & electrolyte disorder	2.3 (2.23,2.37)	<.001	2.3 (2.21,2.4)	<.001	2.24 (2.11,2.39)	<.001	2.3 (2.12,2.5)	<.001	2.36 (2.18,2.56)	<.001
Blood loss anemia	0.8 (0.73,0.88)	<.001	0.77 (0.67,0.89)	<.001	0.82 (0.68,0.99)	<.001	0.92 (0.76,1.1)	0.355	0.7 (0.52,0.93)	0.015
Deficiency anemia	0.95 (0.92,0.99)	0.005	0.97 (0.92,1.02)	<.001	0.94 (0.87,1.02)	0.039	0.96 (0.89,1.03)	0.214	0.93 (0.85,1.01)	0.077
Alcohol abuse	1.18 (1.12,1.23)	<.001	1.2 (1.12,1.28)	0.228	1.12 (1.02,1.23)	0.142	1.19 (1.06,1.34)	0.003	1.11 (0.98,1.27)	0.093

© 2022 Glance LG et al. *JAMA Network Open.*

Drug abuse	0.87 (0.81,0.95)	0.001	0.9 (0.8,1.01)	<.001	0.84 (0.71,0.99)	0.018	0.79 (0.65,0.96)	0.016	0.97 (0.85,1.12)	0.716
Psychoses	0.89 (0.84,0.94)	<.001	0.88 (0.81,0.96)	0.080	0.83 (0.72,0.95)	0.039	0.91 (0.81,1.02)	0.100	0.98 (0.85,1.12)	0.733
Depression	0.84 (0.81,0.87)	<.001	0.83 (0.8,0.87)	0.004	0.85 (0.77,0.92)	0.007	0.85 (0.79,0.92)	<.001	0.83 (0.75,0.9)	<.001
Procedure										
AAA repair	18.6 (15.8,21.9)	<.001	17.5 (14.1,21.7)	<.001	18.8 (12.5,28.3)	<.001	19.8 (13.3,29.5)	<.001	19.6 (11.8,32.5)	<.001
Amputation	1.65 (1.51,1.8)	<.001	1.57 (1.38,1.78)	<.001	1.6 (1.3,1.9)	<.001	1.8 (1.4,2.2)	<.001	2 (1.6,2.4)	<.001
Appendectomy	1.32 (1.16,1.5)	<.001	1.39 (1.14,1.69)	0.001	1.1 (0.8,1.5)	0.478	1.3 (1,1.7)	0.046	1.5 (1.1,2.1)	0.014
Bile duct, liver or pancreas	5.41 (4.86,6.01)	<.001	5.05 (4.3,5.93)	<.001	5.3 (4.1,6.8)	<.001	5.7 (4.7,6.9)	<.001	6.5 (4.8,8.8)	<.001
Cardiac surgery	10.75 (9.69,11.92)	<.001	10 (8.61,11.61)	<.001	11.7 (9.3,14.8)	<.001	11.2 (8.7,14.3)	<.001	11.6 (8.9,15.2)	<.001
CABG	2.92 (2.61,3.27)	<.001	3.04 (2.62,3.52)	<.001	2.8 (2.2,3.7)	<.001	2.8 (2.1,3.7)	<.001	2.6 (1.9,3.7)	<.001
CABG with IMA (no vein)	3.48 (2.82,4.3)	<.001	4.23 (3.28,5.46)	<.001	4.1 (2.8,6.1)	<.001	1.6 (0.9,3)	0.107	3.9 (2.3,6.7)	<.001
Carotid endarterectomy	0.99 (0.81,1.2)	0.882	0.8 (0.6,1.08)	0.147	1.3 (0.9,1.9)	0.227	1.3 (0.9,1.9)	0.220	0.7 (0.3,1.6)	0.413
Cholecystectomy	reference		reference		reference		reference		reference	
Colon surgery	5.71 (5.28,6.17)	<.001	5.62 (5.01,6.3)	<.001	5.5 (4.65,6.5)	<.001	5.74 (4.8,6.85)	<.001	6.36 (5.05,0)	<.001
Craniotomy	12.07 (10.92,13.34)	<.001	11.15 (9.63,12.9)	<.001	13.37 (10.69,16.73)	<.001	11.76 (9.39,14.73)	<.001	14.19 (10.8,0)	<.001
Spinal fusion	1.62 (1.45,1.81)	<.001	1.54 (1.32,1.79)	<.001	1.46 (1.13,1.89)	0.004	1.73 (1.33,2.25)	<.001	2.03 (1.42,0)	<.001
Fracture surgery	0.93 (0.84,1.02)	0.127	0.85 (0.75,0.97)	0.019	0.95 (0.77,1.17)	0.640	0.89 (0.68,1.17)	0.403	1.2 (0.91,0)	0.186
Gastric surgery	4.55 (4.14,5.01)	<.001	4.64 (3.99,5.39)	<.001	4.44 (3.53,5.58)	<.001	4.67 (3.86,5.65)	<.001	4.48 (3.67,0)	<.001
Hip arthroplasty	0.92 (0.83,1.02)	0.116	0.84 (0.73,0.97)	0.014	0.9 (0.71,1.15)	0.400	1.04 (0.82,1.31)	0.776	1.12 (0.85,0)	0.431
Knee arthroplasty	0.4 (0.33,0.47)	<.001	0.34 (0.26,0.44)	<.001	0.32 (0.21,0.49)	<.001	0.51 (0.37,0.71)	<.001	0.58 (0.41,0)	0.003
Laminectomy	1.38 (1.2,1.57)	<.001	1.4 (1.16,1.69)	<.001	0.99 (0.73,1.35)	0.973	1.49 (1.12,1.98)	0.006	1.69 (1.09,0)	0.018
Peripheral vascular bypass	4.17 (3.74,4.66)	<.001	3.86 (3.28,4.54)	<.001	3.4 (2.65,4.37)	<.001	5.05 (4.06,6.29)	<.001	5.4 (4,0)	<.001
Small bowel surgery	4.88 (4.47,5.33)	<.001	4.69 (4.13,5.33)	<.001	4.53 (3.74,5.48)	<.001	5.12 (4.22,6.2)	<.001	5.88 (4.59,0)	<.001
Thoracic surgery	5.56 (5.08,6.08)	<.001	5.11 (4.5,5.8)	<.001	5.5 (4.45,6.79)	<.001	6.32 (5.14,7.77)	<.001	6.37 (4.91,0)	<.001
Exploratory laparotomy	4.49 (4.14,4.86)	<.001	4.26 (3.79,4.78)	<.001	4.59 (3.89,5.4)	<.001	4.91 (4.06,5.94)	<.001	4.62 (3.71,0)	<.001

© 2022 Glance LG et al. *JAMA Network Open.*

**eTable 4.** Model Coefficients for DDD model examining the association between changes in all-cause inpatient mortality and payer status & hospital COVID-19 burden		
	AOR (95% CI)	P
Payer		
Commercial insurance	reference	
Medicare	1.26 (1.17 , 1.35)	<.001
Medicaid	1.27 (1.17 , 1.38)	<.001
Uninsured	1.51 (1.28 , 1.77)	<.001
Other payer	1.32 (1.17 , 1.48)	<.001
Time Period		
Baseline	reference	
Surge	0.91 (0.82 , 1.02)	0.097
Payer X Surge		
Commercial insurance X Surge	reference	
Medicare X Surge	0.95 (0.83 , 1.07)	0.385
Medicaid X Surge	1.03 (0.85 , 1.24)	0.772
Uninsured X Surge	1.22 (0.93 , 1.61)	0.152
Other payer X Surge	1.21 (0.96 , 1.52)	0.107
Hospital COVID Burden		
<= 5%	reference	
5.1-10.0%	0.98 (0.84 , 1.13)	0.754
10.1-25.0%	0.92 (0.82 , 1.05)	0.211
>25%	0.84 (0.71 , 0.99)	0.039
Hospital COVID burden X Surge		
<=5% X Surge	reference	
5.1-10% X Surge	0.96 (0.8 , 1.14)	0.632
10.1-25% X Surge	1.2 (1 , 1.42)	0.045
>25% X Surge	1.33 (1.07 , 1.66)	0.010
Payer X Hospital COVID-19 burden		
Medicare X COVID-19 (5.1-10%)	1.01 (0.91 , 1.12)	0.822
Medicare X COVID-19 (10.1-25%)	1.02 (0.92 , 1.12)	0.738
Medicare X COVID-19 (>25%%)	1.06 (0.94 , 1.18)	0.335
Medicaid X COVID-19 (5.1-10%)	1.06 (0.93 , 1.21)	0.375
Medicaid X COVID-19 (10.1-25%)	0.96 (0.83 , 1.1)	0.518
Medicaid X COVID-19 (>25%%)	1.07 (0.93 , 1.25)	0.347
Uninsured X COVID-19 (5.1-10%)	1.18 (0.89 , 1.56)	0.252
Uninsured X COVID-19 (10.1-25%)	1.6 (1.2 , 2.13)	0.001

© 2022 Glance LG et al. *JAMA Network Open.*

Uninsured X COVID-19 (>25%%)	1.48 (0.91 , 2.4)	0.117
Other payer X COVID-19 (5.1-10%)	1.02 (0.81 , 1.3)	0.841
Other payer X COVID-19 (10.1-25%)	1.24 (1.04 , 1.47)	0.017
Other payer X COVID-19 (>25%%)	1.27 (1.03 , 1.57)	0.027
Payer X Hospital COVID-19 burden X Surge		
Medicare X COVID-19 (5.1-10%)	1.12 (0.9 , 1.39)	0.321
Medicare X COVID-19 (10.1-25%) X Surge	0.93 (0.76 , 1.13)	0.463
Medicare X COVID-19 (>25%%) X Surge	1.04 (0.81 , 1.33)	0.754
Medicaid X COVID-19 (5.1-10%) X Surge	1.16 (0.87 , 1.55)	0.318
Medicaid X COVID-19 (10.1-25%) X Surge	0.98 (0.73 , 1.33)	0.914
Medicaid X COVID-19 (>25%%) X Surge	1.1 (0.79 , 1.51)	0.579
Uninsured X COVID-19 (5.1-10%) X Surge	0.9 (0.57 , 1.42)	0.645
Uninsured X COVID-19 (10.1-25%) X Surge	0.7 (0.37 , 1.35)	0.290
Uninsured X COVID-19 (>25%%) X Surge	1.04 (0.51 , 2.12)	0.909
Other payer X COVID-19 (5.1-10%) X Surge	1.06 (0.74 , 1.5)	0.758
Other payer X COVID-19 (10.1-25%) X Surge	0.97 (0.64 , 1.47)	0.894
Other payer X COVID-19 (>25%%) X Surge	0.89 (0.52 , 1.5)	0.658
Admission status		
Emergent	4.69 (4.41 , 4.99)	<.001
Urgent	4.04 (3.73 , 4.36)	<.001
Elective	reference	
Sex		
Male	reference	
Female	0.94 (0.91 , 0.96)	<.001
Age		
18-30	reference	
31-50	1.12 (1.05 , 1.21)	0.001
51-64	1.49 (1.38 , 1.6)	<.001
65-74	1.81 (1.67 , 1.96)	<.001
75-79	2.14 (1.97 , 2.33)	<.001
80-84	2.38 (2.18 , 2.59)	<.001
85-89	2.67 (2.44 , 2.93)	<.001
90 -	3.36 (3.06 , 3.7)	<.001
Covid positive	5.53 (4.73 , 6.47)	<.001
Comorbidity		
Congestive heart failure	1.88 (1.82 , 1.94)	<.001
Valvular disease	1.29 (1.23 , 1.34)	<.001

© 2022 Glance LG et al. *JAMA Network Open.*

Pulmonary circulation disorder	1.57 (1.45 , 1.7)	<.001
Peripheral vascular disease	1.73 (1.67 , 1.78)	<.001
Hypertension	1.01 (0.98 , 1.05)	0.440
Paralysis	1.19 (1.14 , 1.25)	<.001
Other neurological disorders	1.13 (1.09 , 1.17)	<.001
Chronic pulmonary disease	1.17 (1.13 , 1.21)	<.001
Diabetes, uncomplicated	0.96 (0.92 , 1)	0.060
Diabetes, complicated	1.07 (1.04 , 1.1)	<.001
Hypothyroidism	0.98 (0.96 , 1.01)	0.115
Renal failure	1.61 (1.56 , 1.66)	<.001
Liver disease	1.44 (1.38 , 1.49)	<.001
Peptic ulcer disease	1.43 (1.33 , 1.52)	<.001
AIDS/HIV	1.05 (0.87 , 1.27)	0.604
Lymphoma	1.36 (1.24 , 1.48)	<.001
Metastatic cancer	1.45 (1.39 , 1.52)	<.001
Solid tumor without metastasis	0.93 (0.88 , 0.98)	0.010
Rheumatoid arthritis	1.14 (1.08 , 1.19)	<.001
Coagulopathy	2.53 (2.42 , 2.64)	<.001
Obesity	1.07 (1.04 , 1.11)	<.001
Weight loss	1.4 (1.34 , 1.46)	<.001
Fluid & electrolyte disorder	2.3 (2.23 , 2.37)	<.001
Blood loss anemia	0.8 (0.73 , 0.88)	<.001
Deficiency anemia	0.95 (0.92 , 0.99)	0.006
Alcohol abuse	1.17 (1.12 , 1.23)	<.001
Drug abuse	0.88 (0.81 , 0.95)	0.001
Psychoses	0.89 (0.84 , 0.94)	<.001
Depression	0.84 (0.81 , 0.87)	<.001
Procedure		
AAA repair	18.59 (15.78 , 21.9)	<.001
Amputation	1.65 (1.52 , 1.8)	<.001
Appendectomy	1.32 (1.16 , 1.5)	<.001
Bile duct, liver or pancreas	5.41 (4.86 , 6.03)	<.001
Cardiac surgery	10.8 (9.73 , 11.99)	<.001
CABG	2.93 (2.62 , 3.28)	<.001
CABG with IMA (no vein)	3.51 (2.85 , 4.34)	<.001
Carotid endarterectomy	0.98 (0.81 , 1.2)	0.878
Cholecystectomy	reference	

© 2022 Glance LG et al. *JAMA Network Open.*

Colon surgery	5.71 (5.28 , 6.18)	<.001
Craniotomy	12.07 (10.91 , 13.34)	<.001
Spinal fusion	1.62 (1.45 , 1.82)	<.001
Fracture surgery	0.93 (0.84 , 1.02)	0.122
Gastric surgery	4.57 (4.15 , 5.03)	<.001
Hip arthroplasty	0.92 (0.83 , 1.02)	0.113
Knee arthroplasty	0.4 (0.34 , 0.47)	<.001
Laminectomy	1.38 (1.2 , 1.58)	<.001
Peripheral vascular bypass	4.19 (3.75 , 4.68)	<.001
Small bowel surgery	4.88 (4.47 , 5.33)	<.001
Thoracic surgery	5.57 (5.09 , 6.1)	<.001
Exploratory laparotomy	4.49 (4.15 , 4.86)	<.001

© 2022 Glance LG et al. *JAMA Network Open.*
